# Intake of 12 food groups and disability-adjusted life years from coronary heart disease, stroke, type 2 diabetes, and colorectal cancer in 16 European countries

**DOI:** 10.1007/s10654-019-00523-4

**Published:** 2019-04-27

**Authors:** Lukas Schwingshackl, Sven Knüppel, Nathalie Michels, Carolina Schwedhelm, Georg Hoffmann, Khalid Iqbal, Stefaan De Henauw, Heiner Boeing, Brecht Devleesschauwer

**Affiliations:** 1 Department of Epidemiology, German Institute of Human Nutrition Potsdam-Rehbruecke (DIfE), Arthur-Scheunert-Allee 114-116, 14558 Nuthetal, Germany; 2 NutriAct-Competence Cluster Nutrition Research Berlin-Potsdam, 14458 Nuthetal, Germany; 3 Institute for Evidence in Medicine, Faculty of Medicine and Medical Centre, University of Freiburg, Breisacher Straße 153, 79110 Freiburg, Germany; 4 Department of Public Health, Ghent University, 9000 Ghent, Belgium; 5 Department of Nutritional Sciences, University of Vienna, Althanstraße 14, UZA II, 1090 Vienna, Austria; 6 Department of Human Nutrition, Institute of Basic Medical Sciences, Khyber Medical University, Peshawar, Pakistan; 7 Department of Epidemiology and Public Health, Sciensano, Rue Juliette Wytsman 14, 1050 Brussels, Belgium; 8 Department of Veterinary Public Health and Food Safety, Faculty of Veterinary Medicine, Ghent University, Salisburylaan 133, 9820 Merelbeke, Belgium

**Keywords:** Disability-adjusted life years, Food groups, Comparative risk assessment, Population health-impact, Type 2 diabetes, Coronary heart disease, Stroke, Colorectal cancer

## Abstract

Our aim was to estimate and rank 12 food groups according to disability-adjusted life years (DALYs) from coronary heart disease (CHD), stroke, type 2 diabetes (T2D), and colorectal cancer (CRC) in 16 European countries. De novo published non-linear dose–response meta-analyses of prospective studies (based on 297 primary reports), and food consumption data from the European Food Safety Authority Comprehensive European Food Consumption Database in Exposure Assessment, and DALY estimates from the Institute for Health Metrics and Evaluation were used. By implementing disease-specific counterfactual scenarios of theoretical minimum risk exposure level (TMRELs), the proportion of DALYs attributed to 12 food groups was estimated. In addition, a novel modelling approach was developed to obtain a single (optimized) TMREL across diseases. Four scenarios were analysed (A: disease-specific TMRELs/all food-disease associations; B: disease-specific TMRELs/only significant food-disease associations; C: single TMREL/all food-disease associations; D: single TMREL/only significant food-disease associations). Suboptimal food intake was associated with the following proportions of DALYs; Scenario A (highest-estimate) and D (lowest-estimate): CHD (A: 67%, D: 52%), stroke (A: 49%, D: 30%), T2D (A: 57%, D: 51%), and CRC (A: 54%, D: 40%). Whole grains (10%) had the highest impact on DALYs, followed by nuts (7.1%), processed meat (6.4%), fruit (4.4%) and fish and legumes (4.2%) when combining all scenarios. The contribution to total DALYs of all food groups combined in the different scenarios ranged from 41–52% in Austria to 51–69% in the Czech-Republic. These findings could have important implications for planning future food-based dietary guidelines as a public health nutrition strategy.

**Electronic supplementary material:**

The online version of this article (10.1007/s10654-019-00523-4) contains supplementary material, which is available to authorized users.

## Introduction

According to the Global Burden of Disease (GBD) 2016 study, a suboptimal diet, characterized by low intakes of whole grains, fruits, vegetables, nuts, seafood, fibre, legumes, polyunsaturated fatty acids (PUFA), calcium, milk, and high intakes of salt, trans fatty acids, processed meat, red meat, and sugar sweetened beverages (SSB) is a leading risk factor for premature death and disability worldwide [1].

In view of the now preferred tool of food-based dietary guidelines (FBDGs) to guide populations regarding their dietary habits, more data on foods are needed rather than on nutrients and specific dietary factors [2]. In the past, the population health metrics often used to substantiate FBDGs were limited to incidence or cause-specific mortality based relative risks, without further quantifying the impact or taking disease severity into account [2]. However, ranking food groups according to their health impact is of major interest when developing strategies for improving population health. In this respect the disability-adjusted life year (DALY) has shown to be a suitable and comparable quantifying measure of the burden of disease associated with exposure by combining “years lived with disability” and “years of life lost” [3, 4].

Particularly for a new generation of FBDGs, the impact of these guidelines should be evaluated and potential impacts simulated for the development of effective public health nutrition strategies. We published recently a series of dose–response meta-analyses investigating the association between 12 a priori defined food groups and risk of particularly pronounced nutrition-related chronic diseases (e.g. coronary heart disease (CHD), stroke, type 2 diabetes (T2D), and colorectal cancer (CRC) [2, 5–9] using standardized methodology. The rationale behind the selection of these 12 food groups is that they are the base of most FBDGs and diet quality indices/scores, and have often shown a strong relation with nutrition-related chronic diseases [2, 10].

Often, a non-linear association between these food groups and risk of CHD, stroke, T2D and CRC could be observed [5, 6, 9]. Since previous published comparative risk assessment (CRA) modelling studies evaluating the health impact of dietary risk factors were limited to linear associations from meta-analyses [1, 11], the current investigation aimed to use a CRA design to estimate the association between 12 a priori defined food groups and DALYs due to CHD, stroke, T2D, and CRC by implementing non-linear associations. In addition to using disease-specific theoretical minimal risk exposure levels (TMRELs), we also developed a novel modelling approach to obtain a single TMREL across diseases. Dietary surveys from 16 European countries provided the necessary exposure data. Calculations were also made considering either all or only significant associations. The final aim of the investigation was to obtain a meaningful relative ranking of the different food groups in terms of population health impact.

## Methods

### Study design

A CRA was used to estimate the number of DALYs from CHD, stroke, T2D, and CRC associated with suboptimal intakes (i.e., intakes higher or lower than the optimal consumption level) of 12 food groups in 16 European countries, both per food group and for all food groups combined [1]. The model incorporated separately derived data on (1) dietary habits from the European Food Safety Authority (EFSA) Comprehensive European Food Consumption Database in Exposure Assessment in 16 European countries; (2) the estimated relationships of 12 food groups with CHD, stroke, T2D, and CRC from recent non-linear dose–response meta-analyses of prospective studies [2, 5–9]; (3) the optimal population intake distributions of these food groups based on observed intakes associated with lowest risk in recent meta-analyses [2, 5–9]; and (4) estimated European-specific DALYs from the Institute for Health Metrics and Evaluation (IHME) [12].

### Included food groups and estimated diet-disease relationships

For the present CRA study we used data from our series of recently published linear and non-linear dose–response meta-analyses investigating the association between 12 a priori defined food groups (whole grains, refined grains, vegetables, fruits, nuts, legumes, eggs, dairy, fish, red meat, processed meat, and SSB) and risk of CHD, stroke, T2D, and CRC [5, 6, 9]. For CHD and stroke, T2D, and CRC, 123 reports, 88 reports, and 86 reports were identified respectively. An inverse association was present for whole grains (CHD, T2D, CRC), vegetables (CHD, stroke, T2D, CRC), fruits (CHD, stroke, T2D, CRC), nuts (CHD), legumes (CHD), dairy (CHD, stroke, T2D, and CRC), and fish consumption (CHD, stroke), while a positive association was present for egg (T2D), red meat (stroke, T2D, CRC), processed meat (stroke, T2D, CRC), and SSB consumption (CHD, stroke, T2D) in the non-linear dose–response meta-analyses [5, 6, 9].

The non-linear dose response relationships were fitted in R 3.5.0 using the ‘dosresmeta’ package [13].

### National distributions of dietary intake

Dietary intake data were retrieved from the freely-available EFSA Comprehensive European Food Consumption Database in Exposure Assessment [14]. Dietary intakes were estimated using nationally representative data from the EFSA database including adult populations (excluding those from elderly participants (≥ 65 years of age) and participants < 18 years of age) from 16 European countries (Austria, Belgium, Czech Republic, Denmark, Finland, France, Germany, Hungary, Ireland, Italy, Latvia, the Netherlands, Romania, Spain, Sweden, United Kingdom). Overall, dietary habits from 41,056 participants (from 308 in Austria to 10,419 in Germany) were collected between 1997 and 2012. In eight countries diet was assessed via 3 to 7-day food records, in seven countries via 2*24 h diet recalls, and in one country with a 48 h diet recall (ESM Table 1). Dietary surveys from Slovenia, Slovakia, Poland, Estonia, and Bulgaria were excluded since only a single replication was available.

The average intakes per participant were used to calculate mean values, percentiles, and standard deviations for each food group in each country. The distribution of food group intake in a country reflects the empirical distributions found in the surveys and does not reflect the population distributions to be obtainable by modelling the inter-individual variation [15]. As consequence, the average values were wider compared to modelled population distributions and result in overrepresentation of high and low food consumption intakes. The magnitude of the overrepresentations depends from the number of days recorded per participant which was different between countries.

This database uses the hierarchical FoodEx2 food classification system [14] and the 12 food groups were chosen as follows: vegetables (Level 1: vegetables and vegetable products including fungi), fruits (Level 1: fruits and fruit products), dairy (Level 1: milk and dairy products), eggs (Level 2: eggs, fresh), red meat (Level 2: livestock meat; Level 2: game mammals; Level 2: game birds), processed meat (Level 2: preserved meat; Level 2: sausages; Level 2: pastes, pâtés and terrines; Level 2: meat specialties), sugar sweetened beverages (Level 2: soft drinks), nuts (Level 2: tree nuts), legumes (Level 2: legumes, beans, green, without pods; legumes, beans, dried), fish (Level 1: fish and other seafood), whole grains (all items with ‘whole meal’, ‘bran’ or ‘brown’ on Level 4 from Level 2: grains for human consumption; Level 2: grain milling products; Level 2: bread and rolls; Level 2: breakfast cereals; Level 2: pasta), refined grain (all other non-whole grain items from these latter five ‘Level 2’).

To use the dietary intake data in the subsequent modelling steps, gamma distributions were fitted to the available percentiles using an optimization procedure in R 3.5.0. Specifically, we used the Nelder-Mead algorithm [16], implemented via the “optim” function, to minimize the sum of squared differences between observed and fitted percentiles.

### Characterization of optimal food intakes

Theoretical minimum risk exposure levels (TMRELs) for each food group were characterized (Table 1) based on observed levels associated with lowest disease risk according to non-linear dose–response meta-analysis model of CHD, stroke, T2D, and CRC. In case of monotonically decreasing risk association the mean of P97.5 intake across the 16 considered European countries was used. For all other dose–response models, we obtained the minimum risk exposure level through one dimensional optimization, implemented in R 3.5.0 using the “optimize” function.

**Table 1:** Mean and 97.5 percentile intake of 12 food according to the EFSA database, and theoretical minimum exposure levels (TMREL) from non-linear dose–response meta-analyses including all associations, optimization (single TMREL), and the GBD-2016 study [1], and Micha et al. paper [11] (gram/day)

Food item	Mean consumption EFSA-database [14]	97.5 percentile EFSA-database [14]	TMREL (Optimal intakes)						
			Coronary heart disease [5]	Colorectal cancer [9]	Type 2 diabetes [6]	Stroke [5]	Single (Optimization)	GBD-2016 [1]	Micha et al. 2017 [11]
Refined grain	145	403	65	N/A^b^	**0**	0	60 (0^e^)	N/A	N/A
Whole grain	38	224	**114**	**224** ^**a**^	**56**	124	120 (119^e^)	100–150	125
Vegetables	152	412	**412** ^**a**^	**412** ^**a**^	**282**	**194**	375	290–430	400^d^
Fruit	142	488	**173**	**488** ^**a**^	**260**	**488** ^**a**^	191	200–300	300
Eggs	15	68	0	0	**0**	29	0	N/A	N/A
SSB	120	713	**0**	N/A^b^	**0**	**0**	0	0–5	0
Nuts	2	20	**12**	20^a^	9	9	11	16–25	20.2
Legumes	15	122	**122** ^a^	122^a^	122^a^	122^a^	122^a^	50–70	N/A
Processed meat	44	226	0	**0**	**0**	**0**	0^c^	0–4	0
Red meat	53	195	37	**0**	**0**	**0**	19 (0^e^)	18–27	14.3
Dairy	251	718	**283**	**718** ^**a**^	**718** ^**a**^	**622**	355	350–520	N/A
Fish	26	131	**131** ^**a**^	131^a^	0	**131** ^**a**^	131^a^	N/A	N/A

*GBD* global burden of disease; *EFSA* European food and safety authority; *N/A* not applicable; *SSB* sugar sweetened beverages

^a^Based on maximum consumption; ^b^non-linear RR function not available; ^c^no optimization needed; ^d^including legumes; ^e^optimization TMREL considering only significant associations (scenario D); bold: significant disease-specific associations (95% confidence intervals does not overlap 1)

Although TMRELs per food group and disease will correspond to the highest possible health gains, they are not practical for FBDGs where only one optimal consumption level per food group needs to be proposed. In addition to using disease specific TMRELs, we therefore developed a modelling approach to obtain a single TMREL across diseases. To this end, we used one dimensional optimization, implemented in R 3.5.0 using the “optimize” function, to maximize the sum of DALYs across diseases—thus corresponding to the maximum possible health gain.

### National DALYs

DALYs (mean and 95% uncertainty interval) for CHD, stroke, T2D, and CRC for each of the 16 considered European countries were obtained from the GBD 2016 study [12] via the GBD Results Tool (http://ghdx.healthdata.org/gbd-results-tool) (ESM Table 2). Uncertainty in the DALY estimates was represented by gamma distributions and propagated using 100,000 Monte Carlo simulations.

### DALYs attributable to food groups

We used a CRA approach to calculate disease-specific DALYs attributable to the included food groups. This approach relies on the calculation of a population attributable fraction (PAF), which integrates the exposure distribution with the non-linear dose–response functions, and compares the observed to the counterfactual situation:

$$PAF = \frac{{\mathop \smallint \nolimits_{x = 0}^{n} E\left( x \right)RR\left( x \right)dx - \mathop \smallint \nolimits_{x = 0}^{n} E'\left( x \right)RR\left( x \right)dx}}{{\mathop \smallint \nolimits_{x = 0}^{n} E\left( x \right)RR\left( x \right)dx}}$$

where $E\left( x \right)$ is the observed exposure distribution derived from the EFSA data, $E^{\prime } \left( x \right)$ the counterfactual exposure distribution (TMREL), and $RR\left( x \right)$ the non-linear dose–response function.

To calculate the joint contribution of the 12 food groups, we used a multiplicative approach to combine the food group-specific PAFs [17]:

$$PAF_{comb} = 1 - \mathop \prod \limits_{i} (1 - PAF_{i} )$$

Finally, the attributable DALYs were obtained by multiplying the PAFs (food group-specific or combined) with the corresponding national DALY estimates.

Four different scenarios were implemented, considering (a) either disease-specific or single (optimized) TMRELs, and (b) either all food-disease associations (95% CI overlaps 1) or only significant food-disease associations (95% CI does not overlap 1). The respective scenarios were labelled A (disease-specific TMREL/all food-disease associations); B (disease-specific TMREL/significant food-disease associations); C (single TMREL/all food-disease associations); and D (single TMREL/significant food-disease associations).

An overall “health impact ranking” of the different food groups was obtained by calculating the mean of the attributable DALYs (both absolute values and proportion of total DALYs) across all four scenarios.

## Results

According to the EFSA Comprehensive European Food Consumption Database in Exposure Assessment [14], the mean intake of the 12 food groups across the 16 European countries was 145 g/d for refined grains, 38 g/d for whole grains, 152 g/d for vegetables, 142 g/d for fruits, 2 g/d for nuts, 17 g/d for legumes, 15 g/d for eggs, 251 g/d for dairy, 26 g/d for fish, 53 g/d for red meat, 44 g/d for processed meat, and 120 ml/d for SSB (ESM Table 3–14).

The TMREL (defined as optimal intakes for scenarios A, B, C, and D) for all 12 food groups and the risk of CHD, CRC, T2D, and stroke are shown in Table 1.

### Coronary heart disease

**Fig. 1:**
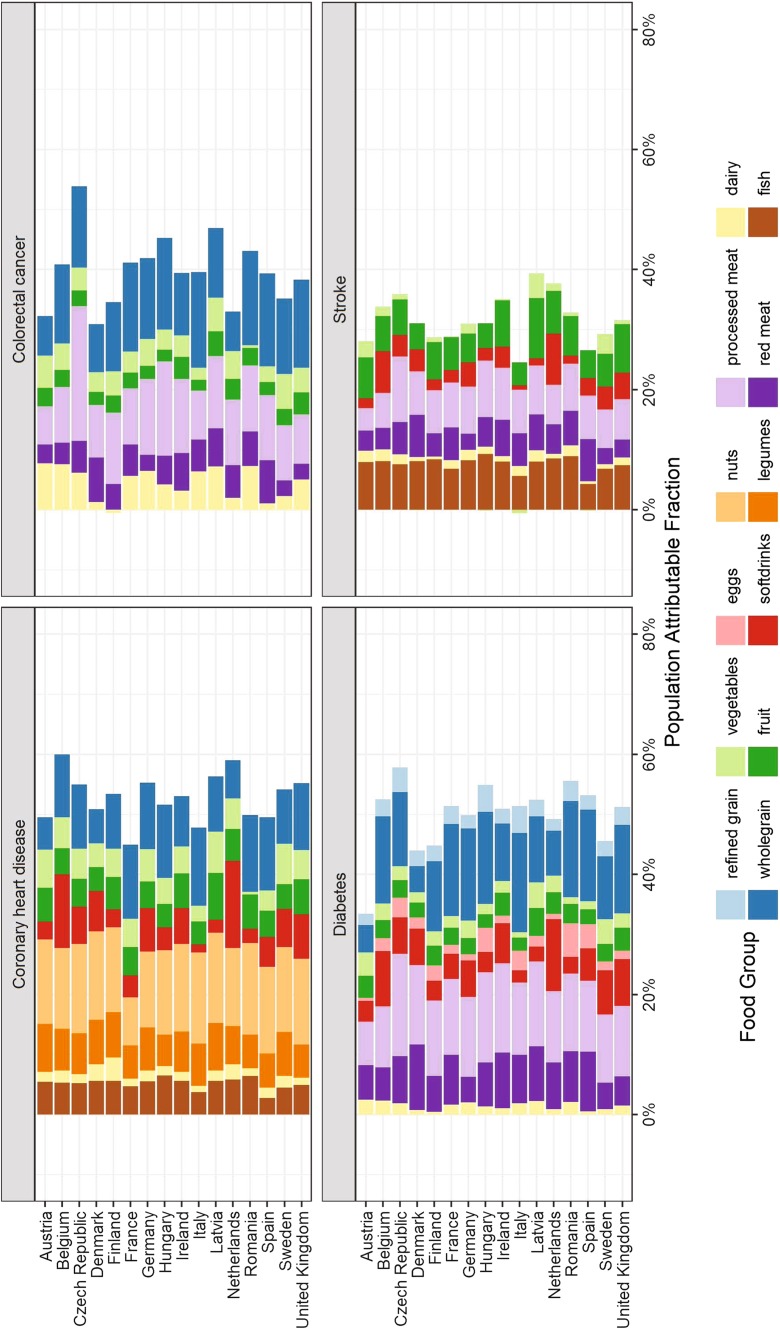
Population attributable fraction for coronary heart disease, colorectal cancer, type 2 diabetes, and stroke associated with suboptimal food intake across 16 European countries for 12 food groups. Analyses based on single theoretical minimum risk exposure levels from combined outcomes and including significant associations (Scenario D)

Overall the suboptimal intake of nuts (18.5%) contributed mostly to the PAF for CHD, followed by whole grains (13.3%), legumes (9%), SSB (7.7%), fish (7%), fruits (6.7%), and vegetable consumption (6.4%) (Fig. 1, ESM Fig. 1–3).

Latvia showed the highest CHD-PAF for nuts (20.6%) and France the lowest (10%). Romania showed the highest PAF for whole grains (16.6%), whereas in Austria and Denmark PAF from whole grains were the lowest (7%). Legumes had the highest PAF in Latvia (10.9%) and the lowest in Hungary (6.9%). SSB-PAFs were highest in the Netherlands (20.4%) and lowest in Italy (1.7%). As for fish consumption, PAF were highest in Hungary (8.6%) and lowest in Spain (3.6%) (ESM Table 15–18).

The 12 food groups were estimated to be associated with an important proportion of DALYs from CHD (A: 6,770,479 (67%) DALYs; B: 5,345,249 (53%) DALYs; C: 6,688,407 (66%) DALYs; D: 5,296,603 (52%) DALYs) (ESM Table 31–34).

### Stroke

Suboptimal intakes of fish (8.9%), processed meat (8.6%), and fruits (7.5%) were the major contributors to the stroke-PAF, followed by red meat (5.1%), legumes (4.4%), SSB (3.8%) and whole grains (3.3%) (Fig. 1, ESM Fig. 1–3).

For fish intake (10.7%), Hungary showed the highest PAF and Spain the lowest (4.8%). For processed meat, the Czech Republic showed the highest PAF (13%) and Austria the lowest (4.3%). Regarding fruit intake, PAF was highest in Latvia (12.1–13.1%) and lowest in Italy (4.2–5.3%) (ESM Table 19–22).

Compared to CHD, the 12 food groups were estimated with a lower proportion of DALYs from stroke (A: 2,459,545 (49%) DALYs; B: 1,674,920 (33%) DALYs; C: 2,088,402 (41%) DALYs; D: 1,530,289 (30%) DALYs) (ESM Table 31–34).

### Type 2 diabetes

Low intakes of whole grains (17.4%) and high intakes of processed meat (16.5%) contributed mostly to the PAF for T2D, followed by suboptimal intakes of red meat (8.7%), SSB (7.2%), fruits (4.4%), and refined grains (3.5%) (Fig. 1, ESM Fig. 1–3).

Italy and Romania showed the highest PAF for whole grains (21.6–24.4%) and the Czech Republic the highest for processed meat (23.6%). For red meat, PAF were highest in Denmark (10.3–13.7%) and lowest in Germany (1.8–5.5%), and SSB-PAF were highest in the Netherlands (15.5%) (ESM Table 23–26).

Similar to CHD, food groups were estimated to be associated with a large proportion of DALYs from T2D (A: 1,589,958 (57%) DALYs; B: 1,495,646 (54%) DALYs; C: 1,404,065 (50%) DALYs; D: 1,415,765 (51%) DALYs) (ESM Table 31–34).

### Colorectal cancer

Suboptimal intake of whole grains (19.2%) and processed meat (13.6%) contributed mostly to the PAF for CRC, followed by dairy (10.3%), red meat (5.6%), vegetables (4.7%) and fruit (3.8%) (Fig. 1, ESM Fig. 1–3).

Italy showed the highest PAF for whole grains (19–26.9%), and the Czech Republic for processed meat (29%), while Austria showed the lowest PAF for both food groups (7.4–16.6%). Regarding red meat, PAF were highest in Spain (7.4–8.6%) and lowest in Sweden (1.8–3%), and dairy PAF were highest in Latvia and Belgium (9.2–18.3%) and lowest in Finland (− 0.6 to 9.5%) (ESM Table 27–30).

The 12 food groups were estimated to be associated with a high proportion of the DALYs from CRC. (A: 1,273,761 (54%) DALYs; B: 1,226,763 (52%) DALYs; C: 1,168,715 (50%) DALYs; D: 949,523 (40%) DALYs) (ESM Table 31–34).

### Total impact and ranking of food groups

An important proportion of total DALYs for CHD, stroke, T2D, and CRC was associated with the 12 food groups (A: 12,093,744 (59%) DALYs; B: 9,742,578 (48%) DALYs; C: 11,349,588 (56%) DALYs; D: 9,192,180 (45%) DALYs) (Table 2).

**Table 2:** Disability-adjusted life years (95% uncertainty interval) attributable to 12 food items in 16 European countries based on the 4 scenario analyses

Country	Disease-specific TMREL All associations (Scenario A)	Disease-specific TMREL Significant associations (Scenario B)	Single TMREL all associations (Scenario C)	Single TMREL Significant associations (Scenario D)	Proportion
Austria	207,229 (195,640–219,199)	176,113 (166,232–186,343)	192,414 (181,496–203,670)	165,522 (155,999–175,383)	47%
Belgium	282,703 (265,375–300,634)	239,063 (224,210–254,454)	266,526 (250,170–283,445)	226,600 (212,250–241,464)	52%
Czech Republic	543,748 (516,978–571,320)	421,000 (399,569–443,184)	521,756 (496,114–548,206)	405,027 (384,288–426,519)	60%
Denmark	127,512 (118,508–136,847)	104,171 (96,795–111,836)	117,382 (108,926–126,181)	95,881 (88,862–103,168)	48%
Finland	163,865 (152,040–176,136)	130,378 (120,803–140,319)	153,483 (142,281–165,093)	123,709 (114,448–133,328)	51%
France	1,179,561 (1,117,898–1,243,006)	946,842 (895,940–999,595)	1,091,083 (1,034,119–1,149,343)	877,747 (829,741–927,513)	48%
Germany	2,734,764 (2,569,376–2,905,901)	2,305,892 (2,164,267–2,452,384)	2,580,159 (2,422,671–2,743,281)	2,192,961 (2,055,578–2,335,109)	54%
Hungary	616,706 (575,581–659,541)	469,186 (438,427–501,146)	586,496 (546,508–628,188)	446,749 (416,785–477,968)	55%
Ireland	96,279 (88,608–104,287)	79,574 (73,229–86,209)	91,067 (83,696–98,752)	75,308 (69,175–81,737)	54%
Italy	1,639,038 (1,542,420–1,739,077)	1,256,809 (1,180,165–1,336,768)	1,528,435 (1,437,801–1,622,275)	1,175,395 (1,102,285–1,251,624)	50%
Latvia	158,323 (146,106–171,139)	125,066 (115,516–135,068)	151,277 (139,405–163,768)	120,953 (111,591–130,792)	58%
Netherlands	380,367 (356,120–405,845)	329,186 (307,663–351,843)	353,840 (331,401–377,391)	306,498 (285,990–328,111)	53%
Romania	1,239,234 (1,162,976–1,318,482)	924,583 (868,377–983,107)	1,165,546 (1,092,791–1,241,549)	882,069 (827,498–938,979)	52%
Spain	949,693 (900,083–1,001,433)	756,903 (715,098–801,104)	885,437 (839,554–933,107)	704,223 (664,608–746,152)	50%
Sweden	269,096 (248,244–290,950)	225,760 (207,925–244,468)	251,088 (231,309–271,729)	212,581 (195,346–230,635)	51%
United Kingdom	1,505,625 (1,434,794–1,578,942)	1,252,051 (1,191,084–1,315,259)	1,413,598 (1,347,220–1,482,312)	1,180,957 (1,122,121–1,241,751)	53%
TOTAL	12,093,744 (11,850,947–12,340,261)	9,742,578 (9,540,578–9,947,540)	11,349,588 (11,119,976–11,583,653)	9,192,180 (8,997,492–9,390,309)	52%
Proportion	59% (55%–65%)	48% (44%–52%)	56% (51%–61%)	45% (42%–49%)	

*TMREL* theoretical minimum risk exposure level

Austria (47%), Denmark (48%), and France (48%) had the lowest mean contribution to the proportion of total DALYs, whereas the highest proportions were observed for the Czech Republic (60%), Latvia (58%), and Hungary (55%) by combining scenario A, B, C, D (Table 2). The disease-specific TMREL values maximize DALYs per outcome (scenario A and B), and thus lead to highest overall DALYs. Taking into account disease-specific TMREL, the total DALYs proportion were lowest for Austria (A: 52%, B: 44%), and the highest for the Czech Republic (A: 69%, B: 53%). The single TMREL generates the single value that maximizes DALYs across outcomes (scenario C and D). Using this approach, the lowest DALYs proportions were again observed for Austria (C: 49%, D: 41%), and the highest for the Czech Republic (C: 66%, D: 51%). By ranking the food groups for the total DALYs, whole grains (10%) had the highest health-impact, followed by nuts (7.1%), processed meat (6.4%), fruits (4.4%), fish (4.2%), legumes (4.2%), and SSB (3.9%) when taking into account all four scenarios (Table 3).

**Table 3:** Disability-adjusted life years attributable to 12 food groups in 16 European countries based on the 4 scenario analyses

Food group	Health impact ranking, Proportion	Disease-specific TMREL All associations (Scenario A)	Disease-specific TMREL Significant associations (Scenario B)	Single TMREL All associations (Scenario C)	Single TMREL Significant associations (Scenario D)
Wholegrain	1 10%	2,149,382 (2,101,443–2,198,486)	2,051,943 (2,001,020–2,103,723)	1,997,744 (1,952,477–2,044,162)	1,879,065 (1,831,945–1,927,466)
Nuts	2 7.1%	1,505,070 (1,471,147–1,540,030)	1,366,224 (1,327,532–1,405,928)	1,490,186 (1,456,137–1,525,291)	1,372,538 (1,334,036–1,411,727)
Processed meat	3 6.4%	1,627,698 (1,592,038–1,663,918)	949,806 (920,396–980,572)	1,677,518 (1,640,408–1,715,219)	981,639 (951,177–1,013,119)
Fruit	4 4.4%	871,116 (854,532–887,939)	980,187 (961,342–999,227)	818,101 (802,323–834,134)	908,337 (890,765–926,085)
Fish	5 4.2%	857,500 (839,760–875,659)	882,500 (862,297–903,083)	812,755 (794,325–831,574)	892,270 (871,985–912,875)
Legumes	6 4.2%	1,018,455 (996,977–1,040,518)	651,384 (632,080–671,307)	1,052,068 (1,029,882–1,074,804)	654,384 (635,023–674,148)
SSB	7 3.9%	734,798 (715,407–754,791)	825,269 (803,612–847,352)	756,512 (736,527–777,112)	833,618 (811,964–855,786)
Red meat	8 3.7%	1,067,779 (1,045,837–1,090,172)	513,665 (498,147–529,598)	847,201 (829,783–865,016)	529,416 (513,453–545,873)
Vegetables	9 3.1%	657,911 (643,665–672,496)	735,626 (719,639–751,972)	539,540 (526,546–552,899)	602,009 (587,606–616,785)
Dairy	10 2.5%	598,562 (586,262–610,922)	642,843 (630,088–655,827)	357,083 (349,768–364,446)	392,300 (384,393–400,305)
Refined grain	11 2.4%	960,979 (937,118–985,421)	82,321 (76,282–88,720)	810,404 (786,904–834,523)	84,049 (77,827–90,577)
Eggs	12 0.4%	44,495 (37,329–51,680)	61,070 (56,557–65,849)	190,475 (183,530–197,587)	62,355 (57,714–67,231)

*SSB* sugar sweetened beverages; *TMREL* theoretical minimum risk exposure level

## Discussion

Suboptimal intakes of 12 major food groups in 16 European countries were associated with 45–59% of total DALYs due to CHD, stroke, T2D, and CRC. CHD was the outcome with the highest proportions of total DALYs (52–67%) associated with food intake, whereas stroke had the lowest proportions (30–49%). The largest estimated DALYs were associated with suboptimal intakes of whole grains (10%), followed by nuts (7.1%), processed meat (6.4%), fruits (4.4%), fish (4.2%), legumes (4.2%), and SSB (3.9%). The DALYs due to suboptimal intake of healthy foods contributed substantially more to the overall health-impact than DALYs related to unhealthy foods. When taking the dietary habits in the 16 European countries, Austria (47%) had the lowest mean contribution to the proportion of total DALYs, whereas the highest proportion was observed for the Czech Republic (60%).

### Comparison with other studies

The calculation of total DALYs and its proportion due to specific exposures depends on the type and number of outcomes and their relation to the exposures including their prevalence in the investigated populations [1]. Thus, it is obvious that the proportion of DALYs due to dietary factors are different between our study (we chose four outcomes strongly associated with suboptimal food intake: CHD, stroke, CRC, T2D) and other studies such as the GBD 2016 analyses of diet-related DALYs that considered ~ 20 different cancer types (most of them not associated with diet), cardiovascular diseases, cerebrovascular diseases, metabolic diseases, asthma, kidney disease, and neurodegenerative diseases in Europe. Comparing the relative contribution of our four outcomes, CHD contributed to 50% of DALYs, followed by stroke (25% DALYs), T2D (14% DALYs), and CRC (11% DALYs). According to IHME, the number of DALYs due to CHD, CRC, T2D, and stroke across the included 16 European countries was 20% of total DALYs. Overall, the IHME considers > 100 causes for the estimation of total DALYs. Thus, in the GBD 2016 study [1], 15 dietary risk factors (foods and nutrients) were associated with total DALYs from 8% (Denmark, France, Netherlands, and Spain) to 18% (Romania). The GBD 2016 study showed for the outcomes CHD, stroke, and T2D that suboptimal intakes of whole grains (range 18–19%), nuts (range 11–21%), and fruits and vegetables (range 8–15%) contributed mainly to DALYs. For US adults, the proportion of death due to CHD, stroke, and T2D based on the associations between 10 dietary factors (fruit, vegetables, nuts/seeds, whole grains, unprocessed red meats, processed meats, SSBs, PUFA, seafood omega-3 fats, and sodium) has been evaluated recently by Micha and co-workers [11]. A suboptimal diet was strongly associated with disease-specific mortality from CHD, stroke, and T2D (range 48–53%), with suboptimal intakes of whole grains (range 4–17%), nuts (range 7–15%), fruits and vegetables (range 6–22%), and processed meat (range 12–18%), representing the main contributors to mortality rates.

We have incorporated non-linear dose–response relationships in our modelling study, whereas the methodological approach of the two other reports was based on published linear dose–response meta-analyses (study-specific slopes: linear trends method described by Greenland and Longnecker) [18]. The use of linear or non-linear models did not result in large differences in the risk estimates (e.g. for whole grains, nuts, legumes, dairy, red meat, processed meat, and SSB). However, the health-impact of fruits and vegetables (range 2–8%) was lower in our analysis compared to those reports (range 6–22%) probably driven by evidence of a significant non-linear dose–response association for CHD (fruits), stroke (vegetables), T2D (fruits and vegetables), and CRC (fruits and vegetables). Based on linear associations, the ranking of a diet low in fruits as the second most important risk factor for stroke (36% DALYs), just after high systolic blood pressure, might be an overestimation [19].

In terms of the major non-communicable diseases (NCDs), the studies, including ours, are consistent with the proportions regarding overall dietary impact and also ranking of foods taking the example of cause-specific mortality in the US. Suboptimal intakes of food were associated with 45–59% of total DALYs in the present study, and between 48 and 53% in the study by Micha and co-workers [11]. Consistently with our study, foods like nuts and whole grains had the highest impact on DALYs/mortality of major NCDs in the studies of Micha et al. [11] /GBD-2016 [1]. It is noteworthy that similar results regarding attributable proportions of DALYs could be obtained by Micha et al. and our study despite the slight differences in how the distributions of the dietary data were calculated.

It is still the question how to deal with significant and non-significant associations. Whereas in the GBD 2016 [1] analysis and also in Micha et al. [11], only dietary factors with a significant association were included, we investigated different scenarios (scenarios A to D). Our scenario approach has the advantage of showing the whole picture including all or only the significant associations. The reasoning of the scenario approach is based on a systematic procedure and includes also the possibility that non-significant associations can become significant or vice versa when adding future data. We are well aware that scenarios with non-significant associations have lower credibility and increased uncertainty compared to scenarios with significant associations only. For example, refined grains contributed to approximately 4–5% of total DALYs (driven by CHD and T2D) due to their high consumption in European countries (mean intake ranged between 82 g/d to 224 g/d) in scenarios A and C (TMREL: 60–65 g/d), despite the association not being significant for CHD (RR_220 g/d_: 1.19, 95% CI: 0.96, 1.49) and being borderline significant for T2D (RR_220 g/d_: 1.06, 95% CI: 1.00, 1.13) [5, 6], but contributed only to 0.5% of total DALYs in scenarios B and D. Overall, the certainty of evidence of a risk-increasing association of refined grains apart from a possible increased risk of T2D (mainly driven by white rice intake in Asia) is low [20].

### Potential implications

The ranking of whole grains as first in terms of population health impact, followed by nuts, may have important implications for planning future FBDGs. For instance, effective community-based strategies (e.g. campaigns, re-formulation, and decreasing taxes) that target improving intakes of whole grains and nuts should be implemented according to these results [21–23]. Moreover, our findings support campaigns like the five-a-day initiative [24], or campaigns reducing SSB [25], and sodium reduction strategies like food re-formulation (e.g. for processed meat, refined grains) [26]. From a public health point of view, fruit and vegetables are considered as one group; by doing so, the suboptimal intake (< 600 g/d) of fruit and vegetables had the 2^nd^ highest impact on DALYs (7.5%). Implementing the findings from a recent meta-analysis, which found further benefits of fruit and vegetable intakes of up to 800 g/d for CHD and stroke [27] in our CRA, would further increase the health impact of fruit and vegetables. Furthermore, while the disease-specific scenarios might be of scientific interest, the scenarios using an optimized intake (by obtaining a single TMREL across CHD, stroke, T2D, and CRC) have practical use since overall recommendations should consider all major disease risks and should not be disease-specific. According to our findings, daily intakes of 4 servings (120 g) of whole grains, ½ serving (11 g) of nuts, 7 servings of fruits and vegetables (550 g), and limiting of SSB and processed meat should be key recommendations.

### Strengths and limitations of this study

Our modelling study has several strengths. The modelling study design incorporated separately derived measures of dietary habits, optimal dietary intakes (TMREL), DALY rates, and estimated food-health relationships. Relative risk functions were based on a series of de novo-published multivariable adjusted non-linear dose–response meta-analyses using standardized methodology. Nationally representative data sets (from repeated dietary recalls and dietary records) on dietary habits from the most harmonized pan-European EFSA database were used for 16 European countries, and DALYs are potentially generalizable to the European population, until more specific data are available. Another major strength is the implementation of four different types of scenario analyses, taking into account disease-specific versus single TMRELs, and all versus only significant associations. To our knowledge, we are the first to propose a joint modelling approach to obtain a single TMREL across disease outcomes.

The study also has limitations that should be considered when translating the results into public health actions. First, the potential of confounding bias (confounding due to insufficient control of other risk factors and also of other food groups) results in a potential overestimation of the risk functions as well the PAF. This phenomenon results subsequently in a potential overestimation of our proportions of DALYs attributed to the 12 food groups. Although we used only the most adjusted risk estimates for each primary study included in the non-linear dose–response analysis, confounding by intake of all other food groups cannot be ruled out, due to the often missing adjustment for other food groups. Also, in the summation of food groups, some overestimation could have occurred due to the intercorrelated nature food intake [28]. Unfortunately, data on intercorrelations are only sparsely available even in the publications of the representative surveys. Most of the primary studies did not adjust for socioeconomic status, limiting our confidence in those risk estimates. On the other side, the main results of the meta-analyses included in the present CRA were confirmed by sensitivity analyses including only studies with a low risk of bias (adjusted for important lifestyle factors: e.g. smoking, physical activity, and BMI) [5, 6, 9]. Second, PAFs rely on the assumption of a causal exposure-outcome association. The assumption of causality needs more than associations including biological plausibility and evidence from intervention trials [29]. Third, the underlying data of our scenarios could change over time. Our results regard not only food-disease associations but also dietary surveys (we used the EFSA database with surveys conducted between 1997 and 2012). For example, the mean consumption of SSB was very low (< 70 ml/d) in several countries (Austria, Finland, France, Italy, Latvia, Romania), whereas recent analyses by the NutriCoDE expert group showed higher intakes of SSB in those countries [30]. Also, the comparability of dietary surveys across countries is limited, mainly because of various survey methodologies, different clustering of age groups, and different use of diverse food categorization systems [14]. We can imagine that even the existing data collections could provide better data on the population distributions when using advanced modelling [15]. Dietary information of the primary studies included in the non-linear dose–response meta-analyses mainly derives from food frequency questionnaires, which results in subjective approximations of past dietary intakes rather than in an assessment of absolute intakes, and is therefore prone to measurement error. Moreover, the diversity of food groups (e.g. whole grain products) is complex and accurate dietary intake measurement in observational studies is difficult to achieve. In rare circumstances single food items and total intake (e.g. whole grains vs. total grain intake) have been combined in the non-linear dose–response meta-analyses, which may drive the optimal intake levels downwards. Finally, we did not cover the whole spectrum of dietary factors [31–33], or diet-related disorders like for instance hypertension, osteoporosis or neurodegenerative disorders, therefore in future studies these endpoints and new dietary exposures should also be taken into account to extend public health actions.

## Conclusion

Twelve pre-defined food groups were estimated to be associated with an important proportion of DALYs from CHD, stroke, T2D, and CRC in 16 European countries. By ranking these food groups, the suboptimal intake of whole grains had the highest impact on DALYs, followed by nuts, processed meat, fruit, fish, legumes, and SSB. These findings could have important implications for planning future FBDGs as a public health nutrition strategy. An important future task would be the incorporation of all food groups (e.g. olive oil, potatoes, chocolate) considered for FBDGs.

## Electronic supplementary material

Below is the link to the electronic supplementary material.

Supplementary material 1 (PDF 1410 kb)
