# Supplementary material for: Intake of 12 food groups and disability-adjusted life years from coronary heart disease, stroke, type 2 diabetes, and colorectal cancer in 16 European countries
Source: Eur J Epidemiol. 2019 Apr 27;34(8):765–75. doi: 10.1007/s10654-019-00523-4 (PMC6602984; doi:10.1007/s10654-019-00523-4)
Supplement: Supplementary file 1 — Supplementary material 1 (PDF 1410 kb) [file 10654_2019_523_MOESM1_ESM.pdf]

Intake of 12 food groups and disability-adjusted life years from coronary heart disease, stroke, type 2 diabetes, and colorectal cancer in 16 European countries

Lukas Schwingshackl[1,2,3], Sven Knüppel[1], Nathalie Michels[4], Carolina Schwedhelm[1,2], Georg Hoffmann[5], Khalid Iqbal[1,2,6], Stefaan De Henauw[4], Heiner Boeing[1,2], Brecht Devleesschauwer[7,8]

[1] Department of Epidemiology, German Institute of Human Nutrition Potsdam-Rehbruecke (DIfE), Arthur-Scheunert-Allee 114-116, 14558 Nuthetal, Germany

[2] NutriAct-Competence Cluster Nutrition Research Berlin-Potsdam, Nuthetal 14458

[3] Institute for Evidence in Medicine, Faculty of Medicine and Medical Centre, University of Freiburg, Breisacher Straße 153, 79110 Freiburg, Germany

[4] Department of Public Health, Ghent University, 9000 Gent, Belgium

[5] Department of Nutritional Sciences, University of Vienna, Althanstraße 14, UZA II, 1090 Vienna, Austria

[6] Department of Human Nutrition, Institute of Basic Medical Sciences, Khyber Medical University, Peshawar, Pakistan

[7] Department of Epidemiology and Public Health, Sciensano, Rue Juliette Wytsman 14, 1050 Brussels, Belgium

[8] Department of Veterinary Public Health and Food Safety, Faculty of Veterinary Medicine, Ghent University, Salisburylaan 133, 9820 Merelbeke, Belgium

Content	Page
ESM Table 1: Dietary surveys included from 16 European countries	3
ESM Table 2: Disability-adjusted life years for coronary heart disease (CHD), colorectal cancer (CRC), type 2 diabetes (T2D), and stroke across 16 European countries	4-5
ESM Table 3-14: Intakes of 12 food groups across 16 European countries	6-17
ESM Figure 1-3: Population attributable fraction for CHD, CRC, T2D, and stroke associated with suboptimal food intake across 16 European countries for 12 food groups	18-20
ESM Table 15-30: Population attributable fraction for CHD, CRC, T2D, and stroke associated with suboptimal food intake across 16 European countries for 12 food groups considering scenarios A, B, C, D	21-36
ESM Table 31-34: Country specific Disability-adjusted life years attributable to 12 food groups in 16 European countries	37-44

ESM Table 1: Dietary surveys included from 16 European countries in the EFSA Comprehensive European Food Consumption Database.

Country	Number	Survey period	Dietary assessment Method
**Austria**	308	2010-2012	1-2*24h recall
**Belgium**	1 292	2004	2*24h recall
**Czech Republic**	1 666	2003-2004	2*24h recall
**Denmark**	1 739<br>2 822	2005-2008<br>2000-2002	7 day food record
**Finland**	1 295<br>1 575	2012<br>2007	48h recall
**France**	2 276	2005-2007	7 day food record
**Germany**	10 419	2005-2007	2*24h recall
**Hungary**	1 074	2003	3 day food record
**Ireland**	1 274<br>958	2008-2010<br>1997-1999	4 day food record<br>7 day food record
**Italy**	2 313	2005-2006	3 day food record
**Latvia**	1 271	2008	2*24h recall
**Netherlands**	2 057<br>750	2007-2010<br>2003	2*24h recall
**Romania**	1 254	2012	7 day food record
**Spain**	410<br>981	2009<br>1999-2001	2*24h recall
**Sweden**	1 430<br>1 210	2010-2011<br>1997-1998	4 day food record<br>7 day food record
**United Kingdom**	1 266<br>1 724	2008-2011<br>2000-2001	4 day food record<br>7 day food record

ESM Table 2: Disability-adjusted life years (DALYs) for coronary heart disease (CHD), colorectal cancer (CRC), type 2 diabetes (T2D), and stroke across 16 European countries.

**Country**	Cause	DALYs (2016)	95% Uncertainty intervals	
**Austria**	CHD	232 227	251 145	216 041
	CRC	38 758	42 769	35 412
	T2D	56 128	66 686	47 040
	Stroke	68 833	77 120	59 863
	Total	395 946	437 720	358 356
**Belgium**	CHD	218 967	238 181	200 345
	CRC	57 283	63 678	51 029
	T2D	61 882	77 610	49 142
	Stroke	116 340	130 062	103 430
	Total	454 472	509 531	403 945
**Czech Republic**	CHD	445 356	471 933	419 289
	CRC	77 847	87 599	70 957
	T2D	101 747	125 277	81 242
	Stroke	165 197	181 357	149 489
	Total	790 147	866 166	720 977
**Denmark**	CHD	96 247	108 228	85 126
	CRC	35 279	39 994	30 969
	T2D	39 552	47 510	32 752
	Stroke	60 016	67 325	53 417
	Total	231 094	263 057	202 264
**Finland**	CHD	149 722	165 926	136 282
	CRC	20 524	23 000	18 320
	T2D	33 863	44 129	25 375
	Stroke	75 047	84 678	66 486
	Total	279 156	317 733	246 464
**France**	CHD	909 823	974 774	842 020
	CRC	329 628	361 798	303 398
	T2D	340 310	407 626	278 799
	Stroke	548 745	601 426	491 599
	Total	2 128 505	2 345 624	1 915 816
**Germany**	CHD	2 474 745	2 684 330	2 262 929
	CRC	486 289	536 583	439 642
	T2D	671 287	815 420	541 588
	Stroke	926 146	1 028 186	817 905
	Total	4 558 468	5 064 519	4 062 064
**Hungary**	CHD	544 040	596 549	489 190
	CRC	100 254	111 398	88 799
	T2D	88 538	107 083	72 333
	Stroke	232 778	257 571	207 864
	Total	965 610	1 072 601	858 186

**Ireland**	CHD	85 756	96 489	76 370
	CRC	20 123	23 160	17 432
	T2D	21 171	26 845	16 201
	Stroke	31 653	35 902	27 546
	Total	158 703	182 396	137 549
**Italy**	CHD	1 311 476	1 429 339	1 211 596
	CRC	334 223	372 932	302 494
	T2D	511 479	614 390	423 133
	Stroke	641 405	712 130	574 753
	Total	2 798 582	3 128 791	2 511 976
**Latvia**	CHD	139 068	155 062	124 114
	CRC	11 828	13 647	10 308
	T2D	15 736	19 293	12 550
	Stroke	73 098	82 141	64 510
	Total	239 731	270 143	211 481
**Netherlands**	CHD	257 200	282 790	236 068
	CRC	105 023	116 166	94 937
	T2D	119 870	151 689	94 411
	Stroke	162 107	178 649	145 787
	Total	644 201	729 293	571 203
**Romania**	CHD	1 041 189	1 138 411	945 287
	CRC	116 837	130 343	104 950
	T2D	102 108	125 823	82 471
	Stroke	776 798	853 437	704 821
	Total	2 036 931	2 248 014	1 837 529
**Spain**	CHD	735 035	782 374	687 816
	CRC	250 804	274 629	229 917
	T2D	260 069	322 346	207 330
	Stroke	389 291	425 747	348 750
	Total	1 635 200	1 805 096	1 473 813
**Sweden**	CHD	238 713	267 393	212 973
	CRC	51 718	58 199	45 742
	T2D	76 921	98 039	60 392
	Stroke	103 126	115 994	90 822
	Total	470 479	539 625	409 929
**United Kingdom**	CHD	1 276 956	1 325 737	1 229 791
	CRC	316 330	330 387	304 061
	T2D	280 893	360 481	217 351
	Stroke	667 392	717 146	615 643
	Total	2 541 571	2 733 751	2 366 846
**Total**	CHD	10 156 520	10 968 662	9 375 239
	CRC	2 352 750	2 586 282	2 148 368
	T2D	2 781 554	3 410 247	2 242 109
	Stroke	5 037 971	5 548 871	4 522 683
	Total	20 328 795	22 514 062	18 288 399

ESM Table 3: Distribution of refined grains (g/d) on averaged individual intakes across 16 European countries.

	Refined grains (g/d)							
**Country**	mean	SD	P5	P10	median	P95	P97.5	P99
**Austria**	87.1	146.1	10.0	50.0	70.0	217.3	295.0	307.5
**Belgium**	132.5	190.2	20.0	36.0	108.4	359.4	422.0	470.7
**Czech Republic**	205.8	107.1	30.7	56.9	158.1	504.9	627.6	765.7
**Denmark**	133.1	99.4	59.6	72.1	124.2	232.6	249.7	289.0
**Finland**	131.4	164.6	25.1	40.0	91.7	324.8	348.8	354.0
**France**	149.2	109.5	18.3	28.7	124.3	364.0	440.0	465.6
**Germany**	127.1	159.6	30.0	46.0	121.5	243.8	244.8	247.2
**Hungary**	223.8	91.7	48.0	73.9	188.0	493.3	537.8	633.7
**Ireland**	139.0	133.5	41.7	58.6	130.6	259.8	264.1	266.3
**Italy**	216.1	107.8	11.6	26.1	179.9	552.6	577.2	628.2
**Latvia**	139.3	143.9	20.0	35.0	120.0	367.5	531.9	693.7
**Netherlands**	81.9	151.8	45.4	65.3	77.5	187.7	236.1	266.7
**Romania**	165.6	89.1	10.7	24.8	122.4	468.7	562.3	705.4
**Spain**	127.1	87.1	8.6	23.8	84.0	338.9	391.4	394.0
**Sweden**	121.9	SD	26.9	38.7	101.2	301.1	322.8	377.4
**United Kingdom**	145.5	146.1	21.1	33.7	113.8	384.1	452.0	531.4

ESM Table 4: Distribution of whole grains (g/d) on averaged individual intakes across 16 European countries.

			Whole grains (g/d)					
**Country**	mean	SD	P5	P10	median	P95	P97.5	P99
**Austria**	89.1	117.7	0.0	0.0	50.0	265.0	330.0	405.0
**Belgium**	52.6	158.4	0.0	0.0	0.0	234.0	362.0	671.0
**Czech Republic**	7.9	22.8	0.0	0.0	0.0	60.0	75.0	112.5
**Denmark**	70.2	72.5	3.1	7.9	49.3	204.4	264.5	336.3
**Finland**	62.0	126.7	0.0	1.3	17.6	267.0	383.6	562.1
**France**	16.7	59.1	0.0	0.0	0.0	88.6	135.7	274.3
**Germany**	42.5	161.2	0.0	0.0	0.0	229.5	332.0	676.3
**Hungary**	4.6	28.7	0.0	0.0	0.0	33.3	51.7	96.0
**Ireland**	57.0	111.5	0.0	0.0	23.2	234.8	341.8	465.4
**Italy**	5.1	36.7	0.0	0.0	0.0	2.7	68.7	136.6
**Latvia**	31.9	81.7	0.0	0.0	0.0	177.5	235.0	347.5
**Netherlands**	93.8	159.2	0.0	0.0	61.3	364.8	504.0	677.0
**Romania**	2.7	15.6	0.0	0.0	0.0	10.7	35.7	67.9
**Spain**	8.1	36.6	0.0	0.0	0.0	49.7	95.8	168.2
**Sweden**	33.9	62.3	0.0	0.0	12.2	138.2	197.5	266.6
**United Kingdom**	24.1	70.1	0.0	0.0	0.0	102.4	163.8	292.2

ESM Table 5: Distribution of vegetables (g/d) on averaged individual intakes across 16 European countries.

	Vegetables (g/d)							
**Country**	mean	SD	P5	P10	median	P95	P97.5	P99
**Austria**	89.5	94.3	0.0	0.0	72.1	252.0	354.0	430.2
**Belgium**	121.5	98.5	0.0	11.4	104.2	301.7	360.1	470.3
**Czech Republic**	125.9	91.0	20.2	31.1	105.0	300.1	351.8	430.2
**Denmark**	166.0	104.6	45.2	62.1	144.3	359.1	444.6	538.2
**Finland**	142.0	107.2	10.9	26.5	120.6	340.5	409.1	484.2
**France**	145.2	87.7	30.1	47.4	132.8	305.7	357.7	427.0
**Germany**	118.0	104.4	0.0	10.0	95.8	314.5	374.9	475.0
**Hungary**	154.7	86.7	35.0	50.5	141.6	310.0	355.9	419.0
**Ireland**	141.8	85.1	33.4	50.6	128.5	300.6	361.0	433.8
**Italy**	234.7	130.4	74.3	100.6	209.9	469.6	535.4	659.7
**Latvia**	89.7	109.5	0.0	0.0	55.0	290.0	390.0	575.0
**Netherlands**	116.3	87.0	0.0	14.2	102.3	278.6	331.5	385.8
**Romania**	381.5	193.5	146.5	180.0	343.9	745.0	854.8	1051.3
**Spain**	208.9	137.5	33.1	56.1	185.7	460.8	544.3	636.9
**Sweden**	67.0	65.3	2.1	7.1	51.8	181.7	216.5	282.2
**United Kingdom**	128.9	90.9	14.8	28.4	112.6	300.2	342.9	398.1

ESM Table 6: Distribution of fruit (g/d) on averaged individual intakes across 16 European countries.

	Fruit (g/d)							
**Country**	mean	SD	P5	P10	median	P95	P97.5	P99
**Austria**	163.6	153.9	0.0	0.0	128.8	431.5	506.5	760.0
**Belgium**	120.3	122.1	0.0	0.0	90.9	351.7	409.7	488.2
**Czech Republic**	124.1	122.5	0.0	0.0	97.0	355.1	430.8	528.3
**Denmark**	174.1	147.3	4.8	19.1	141.2	448.8	549.9	679.9
**Finland**	164.7	178.3	0.0	0.0	119.0	492.7	601.2	819.4
**France**	136.6	132.3	0.0	2.1	110.1	392.1	472.1	579.4
**Germany**	171.4	179.5	0.0	0.0	127.3	510.9	620.6	763.0
**Hungary**	180.5	162.0	0.0	0.0	150.8	473.7	551.7	683.3
**Ireland**	88.4	96.7	0.0	1.9	58.0	286.8	352.0	422.0
**Italy**	189.6	145.9	0.0	11.5	168.7	444.2	550.0	666.2
**Latvia**	108.1	152.3	0.0	0.0	50.0	400.0	510.0	750.0
**Netherlands**	98.5	111.6	0.0	0.0	68.2	325.7	396.3	489.3
**Romania**	153.4	144.3	1.0	17.2	118.1	405.3	516.4	721.4
**Spain**	175.9	161.9	0.0	0.8	144.6	463.8	567.1	707.0
**Sweden**	130.0	113.4	2.1	8.6	108.0	333.6	394.3	480.6
**United Kingdom**	94.8	106.5	0.0	0.0	61.6	293.9	379.8	460.6

ESM Table 7: Distribution of nuts (g/d) on averaged individual intakes across 16 European countries.

				Nuts (g/d)				
**Country**	mean	SD	P5	P10	median	P95	P97.5	P99
**Austria**	4.4	18.9	0.0	0.0	0.0	20.0	40.0	60.0
**Belgium**	1.4	7.1	0.0	0.0	0.0	8.5	25.0	36.0
**Czech Republic**	1.3	7.3	0.0	0.0	0.0	3.9	17.1	40.0
**Denmark**	1.3	4.6	0.0	0.0	0.0	7.1	12.9	23.6
**Finland**	1.8	7.7	0.0	0.0	0.0	12.5	21.7	39.0
**France**	1.1	4.3	0.0	0.0	0.0	6.6	13.8	25.0
**Germany**	3.0	11.1	0.0	0.0	0.0	20.0	35.0	50.0
**Hungary**	2.5	8.8	0.0	0.0	0.0	14.3	24.0	40.0
**Ireland**	1.1	3.6	0.0	0.0	0.0	6.0	10.3	18.4
**Italy**	1.1	4.8	0.0	0.0	0.0	6.7	13.4	21.3
**Latvia**	1.2	8.1	0.0	0.0	0.0	0.0	20.0	50.0
**Netherlands**	1.9	7.8	0.0	0.0	0.0	15.0	27.5	42.5
**Romania**	0.8	4.0	0.0	0.0	0.0	5.7	8.6	17.1
**Spain**	2.0	6.8	0.0	0.0	0.0	13.3	19.1	34.7
**Sweden**	1.4	5.7	0.0	0.0	0.0	7.5	15.5	24.6
**United Kingdom**	1.2	5.5	0.0	0.0	0.0	6.5	13.1	22.9

ESM Table 8: Distribution of legumes (g/d) on averaged individual intakes across 16 European countries.

Legumes (g/d)								
**Country**	mean	SD	P5	P10	median	P95	P97.5	P99
**Austria**	4.5	25.3	0.0	0.0	0.0	5.0	81.0	139.5
**Belgium**	11.7	46.1	0.0	0.0	0.0	68.2	137.1	223.5
**Czech Republic**	7.2	22.0	0.0	0.0	0.0	51.4	60.0	74.0
**Denmark**	10.4	19.8	0.0	0.0	3.7	44.1	59.5	89.6
**Finland**	6.1	20.5	0.0	0.0	0.0	39.9	56.4	93.7
**France**	34.3	44.4	0.0	0.0	17.6	120.5	148.7	192.9
**Germany**	8.8	41.4	0.0	0.0	0.0	63.5	131.6	205.0
**Hungary**	23.0	51.2	0.0	0.0	1.7	161.7	183.3	231.0
**Ireland**	16.4	33.2	0.0	0.0	3.1	76.3	104.5	148.7
**Italy**	17.6	50.7	0.0	0.0	0.0	121.8	146.6	245.2
**Latvia**	2.6	25.0	0.0	0.0	0.0	0.0	0.0	75.0
**Netherlands**	16.5	52.4	0.0	0.0	0.0	116.2	166.6	252.0
**Romania**	30.9	49.1	0.0	0.0	5.7	133.7	167.1	206.6
**Spain**	36.0	74.7	0.0	0.0	0.0	170.2	249.1	307.8
**Sweden**	6.4	24.6	0.0	0.0	0.0	44.2	73.0	108.0
**United Kingdom**	34.2	57.8	0.0	0.0	7.8	148.8	185.1	253.0

ESM Table 9: Distribution of eggs (g/d) on averaged individual intakes across 16 European countries.

				Eggs (g/d)				
**Country**	mean	SD	P5	P10	median	P95	P97.5	P99
**Austria**	3.4	10.3	0.0	0.0	0.0	30.0	30.0	37.5
**Belgium**	10.4	22.4	0.0	0.0	0.0	61.5	75.1	95.0
**Czech Republic**	19.5	25.6	0.0	0.0	8.9	73.9	93.0	111.3
**Denmark**	16.6	14.6	1.4	2.5	13.0	44.8	54.8	66.9
**Finland**	13.7	23.8	0.0	0.0	3.9	60.9	83.2	115.1
**France**	14.8	15.4	0.0	0.0	9.6	45.5	52.5	65.6
**Germany**	7.1	16.4	0.0	0.0	0.0	36.5	60.0	65.0
**Hungary**	26.4	24.3	2.0	3.3	18.7	73.9	90.0	117.6
**Ireland**	11.3	16.1	0.0	0.0	3.9	42.9	54.2	70.0
**Italy**	21.3	24.9	0.0	0.0	13.7	68.6	84.1	102.8
**Latvia**	8.8	22.1	0.0	0.0	0.0	50.0	75.0	100.0
**Netherlands**	5.9	14.1	0.0	0.0	0.0	34.7	50.0	62.5
**Romania**	35.4	26.3	2.1	7.6	30.1	83.7	96.1	122.1
**Spain**	26.5	24.6	0.0	0.0	22.9	73.9	79.4	92.7
**Sweden**	12.0	17.9	0.0	0.0	3.6	48.9	56.5	77.7
**United Kingdom**	12.1	23.2	0.0	0.0	6.0	43.2	59.9	75.6

ESM Table 10: Distribution of dairy products (g/d) on averaged individual intakes across 16 European countries.

	Dairy products (g/d)							
**Country**	mean	SD	P5	P10	median	P95	P97.5	P99
**Austria**	150.3	138.5	10.0	20.0	118.8	390.0	528.5	630.5
**Belgium**	174.1	176.8	0.0	16.2	130.3	492.6	594.0	881.8
**Czech Republic**	171.7	166.6	8.3	22.5	130.5	489.9	587.4	717.8
**Denmark**	370.1	275.2	58.6	88.5	312.0	875.7	1030.5	1269.2
**Finland**	447.3	324.4	51.0	96.4	382.1	1052.1	1244.2	1502.3
**France**	200.7	155.5	29.3	47.1	160.9	499.6	570.4	665.8
**Germany**	191.3	184.1	10.0	21.3	142.5	541.1	662.1	867.9
**Hungary**	260.1	200.1	28.7	46.7	225.8	618.3	744.7	943.3
**Ireland**	286.3	197.8	49.6	81.3	245.1	648.8	789.9	918.5
**Italy**	185.6	121.3	23.3	41.2	173.0	394.4	444.8	504.5
**Latvia**	143.4	143.9	0.0	10.0	105.0	420.0	560.0	660.0
**Netherlands**	357.8	285.7	19.8	44.0	308.6	879.1	1036.7	1277.9
**Romania**	148.6	98.0	22.9	36.8	133.0	328.7	380.2	458.3
**Spain**	363.5	200.5	84.5	140.0	340.0	687.4	809.2	948.1
**Sweden**	331.2	224.4	38.8	86.7	288.7	746.3	881.0	1062.3
**United Kingdom**	230.9	163.8	34.0	59.6	201.6	522.6	627.8	798.4

ESM Table 11: Distribution of fish (g/d) on averaged individual intakes across 16 European countries.

	Fish (g/d)							
**Country**	mean	SD	P5	P10	median	P95	P97.5	P99
**Austria**	15.6	36.3	0.0	0.0	0.0	99.5	125.0	142.5
**Belgium**	25.4	43.9	0.0	0.0	0.0	112.5	147.7	200.8
**Czech republic**	16.7	41.4	0.0	0.0	0.0	112.5	150.0	180.0
**Denmark**	19.5	20.3	0.0	0.1	14.2	57.3	70.3	87.9
**Finland**	28.1	48.4	0.0	0.0	0.0	122.8	152.7	221.4
**France**	30.2	27.7	0.0	0.0	25.1	86.0	98.3	114.6
**Germany**	17.1	39.5	0.0	0.0	0.0	99.5	140.4	175.0
**Hungary**	8.8	27.5	0.0	0.0	0.0	66.7	100.0	133.3
**Ireland**	22.4	30.1	0.0	0.0	9.4	81.0	102.4	135.4
**Italy**	46.4	52.2	0.0	0.0	35.4	150.0	182.1	215.2
**Latvia**	18.3	39.2	0.0	0.0	0.0	100.0	135.0	180.0
**Netherlands**	11.4	29.7	0.0	0.0	0.0	76.7	104.9	139.0
**Romania**	17.7	32.8	0.0	0.0	0.0	78.6	114.3	153.9
**Spain**	69.9	62.9	0.0	2.0	55.8	188.7	231.8	276.6
**Sweden**	34.7	36.7	0.0	0.0	25.0	104.3	132.4	167.9
**United Kingdom**	26.7	31.5	0.0	0.0	18.5	86.4	102.1	135.5

ESM Table 12: Distribution of red meat (g/d) on averaged individual intakes across 16 European countries.

				Red meat (g/d)				
**Country**	mean	SD	P5	P10	median	P95	P97.5	P99
**Austria**	26.7	59.5	0.0	0.0	0.0	130.5	172.0	172.5
**Belgium**	42.8	65.9	0.0	0.0	19.1	146.8	181.4	248.8
**Czech Republic**	57.8	78.2	0.0	0.0	46.9	176.3	219.2	287.8
**Denmark**	76.8	47.2	17.7	26.7	68.8	164.5	192.4	227.0
**Finland**	42.8	76.6	0.0	0.0	23.0	169.0	236.7	337.1
**France**	62.7	49.1	5.5	15.0	54.4	141.8	173.0	229.1
**Germany**	32.6	62.5	0.0	0.0	0.0	136.5	172.5	253.1
**Hungary**	60.6	56.3	0.0	0.0	51.3	147.2	183.3	253.3
**Ireland**	68.8	60.3	0.0	4.2	58.1	178.6	212.5	259.2
**Italy**	61.7	54.9	0.0	0.0	50.7	156.2	187.7	230.4
**Latvia**	64.3	84.5	0.0	0.0	50.0	210.0	252.5	340.0
**Netherlands**	51.8	73.6	0.0	0.0	21.5	191.8	231.0	278.8
**Romania**	63.6	55.9	0.0	0.0	53.6	171.9	203.6	248.3
**Spain**	74.7	69.0	0.0	0.0	65.0	197.5	238.8	282.5
**Sweden**	36.4	47.0	0.0	0.0	25.3	101.8	153.4	206.9
**United Kingdom**	31.8	37.8	0.0	0.0	23.4	98.9	117.6	143.7

ESM Table 13: Distribution of processed meat (g/d) on averaged individual intakes across 16 European countries.

Processed meat (g/d)								
**Country**	mean	SD	P5	P10	median	P95	P97.5	P99
**Austria**	30.3	70.1	0.0	0.0	0.0	115.3	190.0	288.5
**Belgium**	36.3	70.1	0.0	0.0	8.0	166.5	222.5	325.4
**Czech Republic**	80.1	139.2	0.0	0.0	25.0	377.2	468.3	625.2
**Denmark**	31.9	39.3	0.3	0.9	19.0	107.2	134.4	171.7
**Finland**	45.1	85.3	0.0	0.0	5.1	193.0	258.0	352.8
**France**	37.6	49.9	0.0	0.0	22.6	135.2	164.9	232.2
**Germany**	49.8	79.6	0.0	0.0	21.0	200.0	273.0	367.5
**Hungary**	71.7	94.8	0.0	0.0	43.3	241.0	304.3	435.0
**Ireland**	50.9	60.2	0.0	1.9	30.1	169.8	207.4	272.9
**Italy**	29.9	42.6	0.0	0.0	13.4	113.9	140.7	181.3
**Latvia**	45.5	74.5	0.0	0.0	20.0	190.0	240.0	310.0
**Netherlands**	39.0	68.8	0.0	0.0	10.9	173.4	231.1	318.4
**Romania**	44.0	63.6	0.0	0.0	25.4	164.3	204.3	283.6
**Spain**	48.9	69.7	0.0	0.0	26.2	166.9	226.6	321.6
**Sweden**	40.6	58.9	0.0	0.0	20.0	137.5	193.2	266.2
**United Kingdom**	30.2	46.0	0.0	0.0	13.0	112.8	153.2	188.9

ESM Table 14: Distribution of sugar sweetened beverages (ml/d) on averaged individual intakes across 16 European countries.

	Sugar sweetened beverages (ml/d)							
**Country**	mean	SD	P5	P10	median	P95	P97.5	P99
**Austria**	59.9	173.4	0.0	0.0	0.0	330.0	500.0	750.0
**Belgium**	272.9	425.3	0.0	0.0	124.4	1050.0	1410.0	2025.0
**Czech Republic**	109.1	230.8	0.0	0.0	0.0	600.0	750.0	1000.0
**Denmark**	162.6	259.3	0.0	0.0	64.3	650.0	838.0	1301.2
**Finland**	55.5	166.5	0.0	0.0	0.0	345.0	530.0	797.5
**France**	63.4	156.3	0.0	0.0	0.0	354.3	507.1	664.3
**Germany**	120.0	295.8	0.0	0.0	0.0	700.0	1000.0	1450.0
**Hungary**	96.0	192.7	0.0	0.0	0.0	433.3	600.0	1000.0
**Ireland**	123.5	199.7	0.0	0.0	37.6	509.1	708.4	838.5
**Italy**	27.8	75.7	0.0	0.0	0.0	163.3	220.0	330.0
**Latvia**	48.5	127.6	0.0	0.0	0.0	250.0	430.0	625.0
**Netherlands**	341.8	404.7	0.0	0.0	199.1	1154.2	1358.3	1948.5
**Romania**	49.7	143.1	0.0	0.0	0.0	250.0	425.0	571.4
**Spain**	94.2	186.9	0.0	0.0	0.0	465.8	629.2	795.5
**Sweden**	141.1	225.3	0.0	0.0	43.8	527.0	712.5	832.5
**United Kingdom**	151.7	232.1	0.0	0.0	63.2	603.8	794.0	956.9

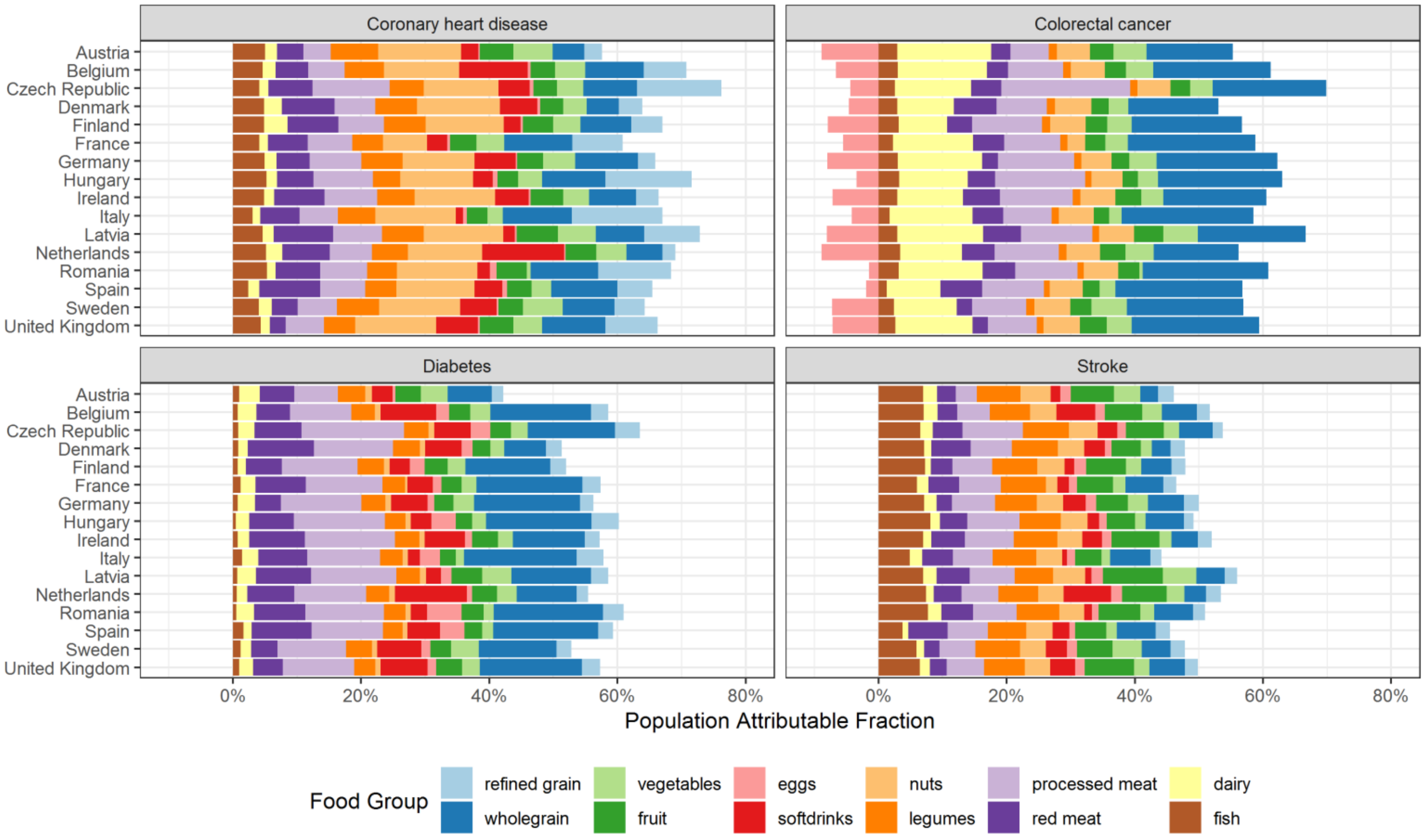

ESM Figure 1: Population attributable fraction for coronary heart disease, colorectal cancer, type 2 diabetes, and stroke associated with suboptimal food intake across 16 European countries for 12 food groups. Analyses based on theoretical minimum risk exposure level from disease-specific outcomes and including all associations (Scenario A).

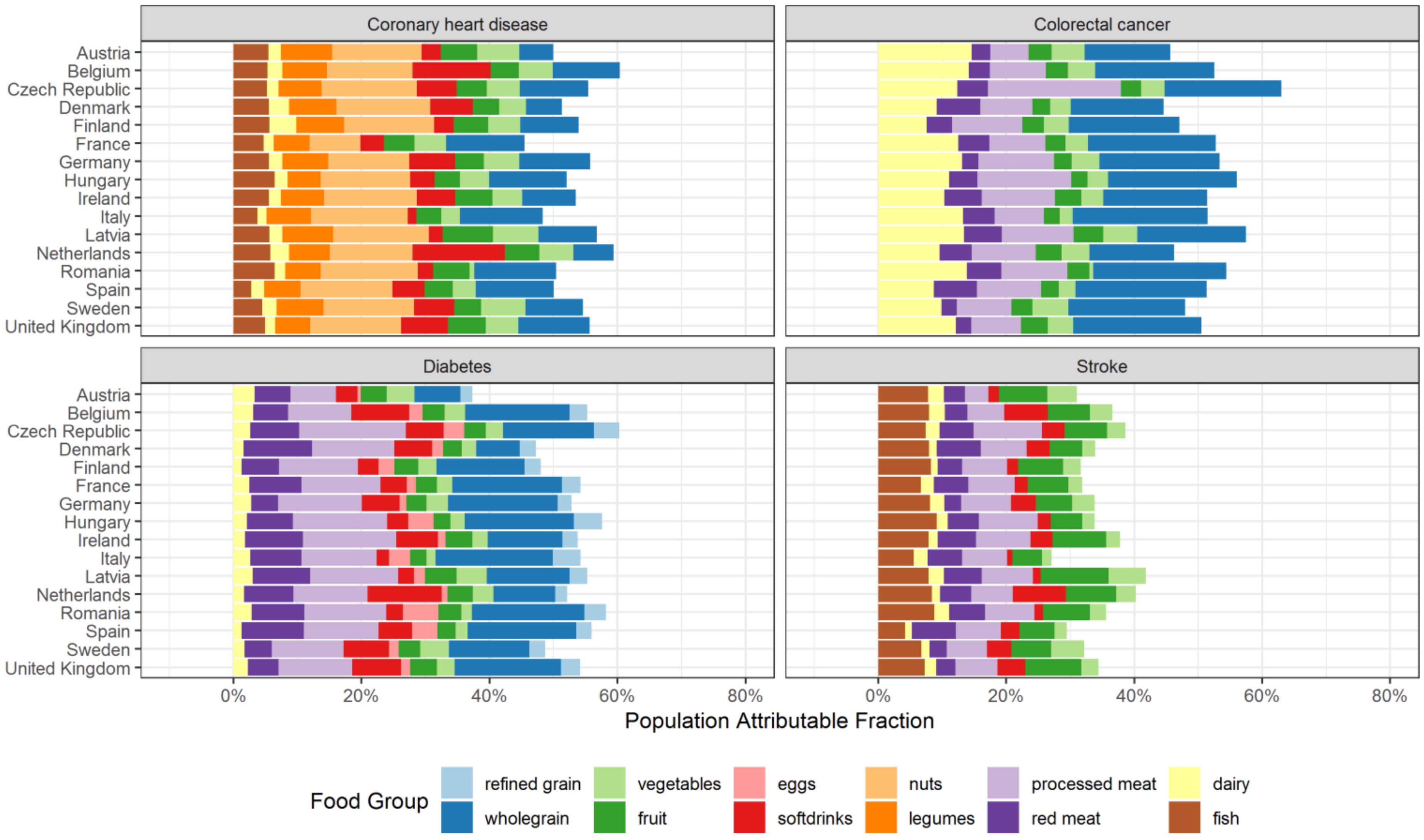

ESM Figure 2: Population attributable fraction for coronary heart disease, colorectal cancer, type 2 diabetes, and stroke associated with suboptimal food intake across 16 European countries for 12 food groups. Analyses based on theoretical minimum risk exposure level from disease-specific and including significant associations (Scenario B).

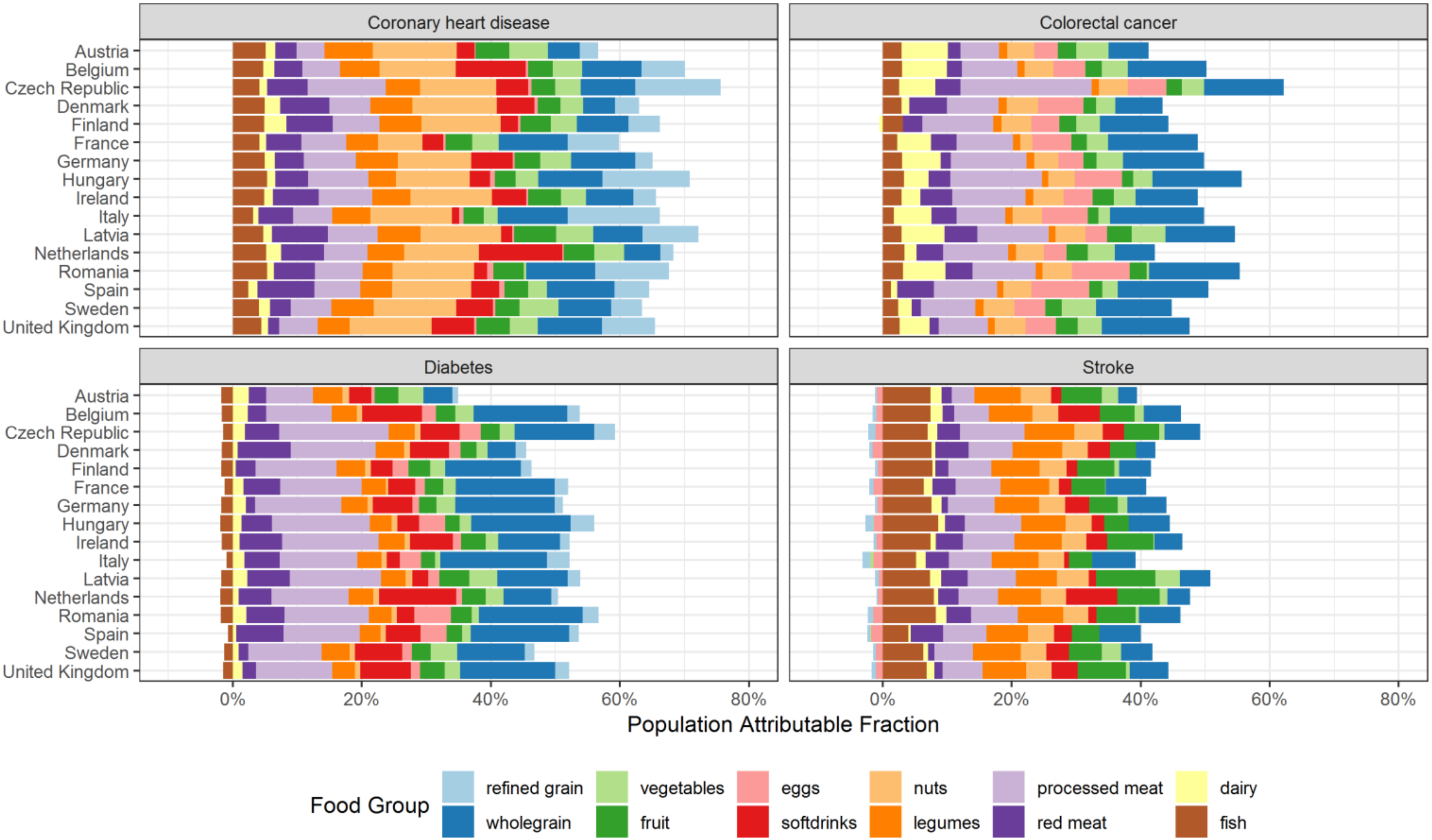

ESM Figure 3: Population attributable fraction for coronary heart disease, colorectal cancer, type 2 diabetes, and stroke associated with suboptimal food intake across 16 European countries for 12 food groups. Analyses based on single theoretical minimum risk exposure level from combined outcomes and including all associations (Scenario C).

ESM Table 15: Population attributable fractions for 12 food groups and coronary heart disease. Analyses based on theoretical minimum risk exposure level from disease-specific outcomes and including all associations (Scenario A).

Country	Refined grain	Whole grain	Vegetables	Fruit	Eggs	SSB	Nuts	Legumes	Processed meat	Red meat	Dairy	Fish	Total
Austria	0.040	0.070	0.087	0.075	0.003	0.039	0.183	0.105	0.060	0.059	0.026	0.071	0.576
Belgium	0.109	0.149	0.076	0.062	0.007	0.175	0.191	0.099	0.093	0.082	0.033	0.076	0.708
Czech Republic	0.228	0.146	0.071	0.064	0.010	0.084	0.203	0.092	0.208	0.119	0.025	0.071	0.762
Denmark	0.055	0.075	0.056	0.054	0.007	0.088	0.195	0.098	0.095	0.124	0.041	0.074	0.638
Finland	0.075	0.123	0.067	0.073	0.006	0.041	0.190	0.101	0.111	0.123	0.057	0.076	0.670
France	0.115	0.156	0.063	0.061	0.006	0.047	0.100	0.071	0.102	0.090	0.020	0.060	0.608
Germany	0.041	0.152	0.076	0.062	0.004	0.099	0.173	0.099	0.124	0.080	0.029	0.076	0.659
Hungary	0.219	0.161	0.060	0.053	0.011	0.050	0.185	0.069	0.152	0.093	0.026	0.086	0.715
Ireland	0.055	0.113	0.063	0.078	0.005	0.081	0.195	0.090	0.127	0.121	0.025	0.075	0.664
Italy	0.217	0.165	0.037	0.049	0.010	0.017	0.192	0.089	0.092	0.094	0.018	0.048	0.670
Latvia	0.144	0.126	0.098	0.108	0.005	0.029	0.206	0.109	0.127	0.155	0.028	0.078	0.728
Netherlands	0.032	0.089	0.076	0.075	0.004	0.204	0.183	0.090	0.105	0.117	0.041	0.082	0.691
Romania	0.178	0.166	0.010	0.074	0.015	0.031	0.197	0.073	0.115	0.110	0.021	0.083	0.684
Spain	0.082	0.158	0.047	0.057	0.012	0.065	0.186	0.074	0.107	0.145	0.026	0.036	0.654
Sweden	0.072	0.122	0.095	0.057	0.005	0.085	0.192	0.099	0.093	0.060	0.031	0.061	0.643
United Kingdom	0.126	0.152	0.069	0.081	0.005	0.100	0.195	0.075	0.091	0.039	0.022	0.067	0.663

SSB: sugar sweetened beverages.

ESM Table 16: Population attributable fractions for 12 food groups and coronary heart disease. Analyses based on theoretical minimum risk exposure level from disease-specific outcomes and including significant associations (Scenario B).

Country	Refined grain	Whole grain	Vegetables	Fruit	Eggs	SSB	Nuts	Legumes	Processed meat	Red meat	Dairy	Fish	Total
Austria	NA	0.070	0.087	0.075	NA	0.039	0.183	0.105	NA	NA	0.026	0.071	0.500
Belgium	NA	0.149	0.076	0.062	NA	0.175	0.191	0.099	NA	NA	0.033	0.076	0.604
Czech Republic	NA	0.146	0.071	0.064	NA	0.084	0.203	0.092	NA	NA	0.025	0.071	0.554
Denmark	NA	0.075	0.056	0.054	NA	0.088	0.195	0.098	NA	NA	0.041	0.074	0.513
Finland	NA	0.123	0.067	0.073	NA	0.041	0.190	0.101	NA	NA	0.057	0.076	0.539
France	NA	0.156	0.063	0.061	NA	0.047	0.100	0.071	NA	NA	0.020	0.060	0.455
Germany	NA	0.152	0.076	0.062	NA	0.099	0.173	0.099	NA	NA	0.029	0.076	0.557
Hungary	NA	0.161	0.060	0.053	NA	0.050	0.185	0.069	NA	NA	0.026	0.086	0.521
Ireland	NA	0.113	0.063	0.078	NA	0.081	0.195	0.090	NA	NA	0.025	0.075	0.535
Italy	NA	0.165	0.037	0.049	NA	0.017	0.192	0.089	NA	NA	0.018	0.048	0.483
Latvia	NA	0.126	0.098	0.108	NA	0.029	0.206	0.109	NA	NA	0.028	0.078	0.568
Netherlands	NA	0.089	0.076	0.075	NA	0.204	0.183	0.090	NA	NA	0.041	0.082	0.594
Romania	NA	0.166	0.010	0.074	NA	0.031	0.197	0.073	NA	NA	0.021	0.083	0.504
Spain	NA	0.158	0.047	0.057	NA	0.065	0.186	0.074	NA	NA	0.026	0.036	0.500
Sweden	NA	0.122	0.095	0.057	NA	0.085	0.192	0.099	NA	NA	0.031	0.061	0.546
United Kingdom	NA	0.152	0.069	0.081	NA	0.100	0.195	0.075	NA	NA	0.022	0.067	0.556

SSB: sugar sweetened beverages, NA: not applicable.

ESM Table 17: Population attributable fractions for 12 food groups and coronary heart disease. Analyses based on single theoretical minimum risk exposure level from combined outcomes and including all associations (Scenario C).

**Country**	**Refined grain**	**Whole grain**	**Vegetables**	**Fruit**	**Eggs**	**SSB**	**Nuts**	**Legumes**	**Processed meat**	**Red meat**	**Dairy**	**Fish**	**Total**
Austria	0.039	0.069	0.083	0.073	0.003	0.039	0.182	0.105	0.060	0.047	0.021	0.071	0.566
Belgium	0.108	0.149	0.073	0.061	0.007	0.175	0.191	0.099	0.093	0.070	0.028	0.076	0.701
Czech Republic	0.228	0.145	0.067	0.063	0.010	0.084	0.203	0.092	0.208	0.108	0.021	0.071	0.756
Denmark	0.055	0.074	0.052	0.053	0.007	0.088	0.194	0.098	0.095	0.113	0.036	0.074	0.630
Finland	0.075	0.123	0.063	0.072	0.006	0.041	0.190	0.101	0.111	0.112	0.052	0.076	0.662
France	0.115	0.156	0.059	0.059	0.006	0.047	0.100	0.071	0.102	0.079	0.015	0.060	0.599
Germany	0.041	0.151	0.073	0.061	0.004	0.099	0.173	0.099	0.124	0.068	0.024	0.076	0.651
Hungary	0.219	0.161	0.057	0.052	0.011	0.050	0.185	0.069	0.152	0.081	0.021	0.085	0.709
Ireland	0.055	0.113	0.060	0.077	0.005	0.081	0.195	0.090	0.127	0.109	0.020	0.075	0.656
Italy	0.217	0.165	0.033	0.048	0.010	0.017	0.192	0.089	0.092	0.082	0.013	0.048	0.662
Latvia	0.144	0.126	0.095	0.107	0.005	0.029	0.206	0.109	0.127	0.144	0.023	0.078	0.722
Netherlands	0.032	0.089	0.072	0.074	0.004	0.204	0.183	0.090	0.105	0.105	0.036	0.082	0.683
Romania	0.178	0.165	0.006	0.073	0.015	0.031	0.196	0.073	0.115	0.098	0.016	0.083	0.676
Spain	0.082	0.158	0.044	0.056	0.012	0.065	0.186	0.074	0.107	0.134	0.021	0.036	0.646
Sweden	0.072	0.122	0.091	0.056	0.005	0.085	0.191	0.099	0.093	0.048	0.026	0.061	0.634
United Kingdom	0.125	0.152	0.066	0.080	0.005	0.100	0.195	0.075	0.091	0.026	0.017	0.067	0.655

SSB: sugar sweetened beverages.

ESM Table 18: Population attributable fractions for 12 food groups and coronary heart disease. Analyses based on single theoretical minimum risk exposure level from combined outcomes and including significant associations (Scenario D).

Country	Refined grain	Whole grain	Vegetables	Fruit	Eggs	SSB	Nuts	Legumes	Processed meat	Red meat	Dairy	Fish	Total
Austria	NA	0.069	0.083	0.073	NA	0.039	0.183	0.105	NA	NA	0.021	0.071	0.495
Belgium	NA	0.149	0.073	0.061	NA	0.175	0.191	0.099	NA	NA	0.028	0.076	0.600
Czech Republic	NA	0.146	0.067	0.063	NA	0.084	0.203	0.092	NA	NA	0.021	0.071	0.550
Denmark	NA	0.074	0.052	0.053	NA	0.088	0.195	0.098	NA	NA	0.036	0.074	0.509
Finland	NA	0.123	0.063	0.072	NA	0.041	0.190	0.101	NA	NA	0.052	0.076	0.534
France	NA	0.156	0.059	0.059	NA	0.047	0.100	0.071	NA	NA	0.015	0.060	0.449
Germany	NA	0.151	0.073	0.061	NA	0.099	0.173	0.099	NA	NA	0.024	0.076	0.553
Hungary	NA	0.161	0.057	0.052	NA	0.050	0.185	0.069	NA	NA	0.021	0.086	0.516
Ireland	NA	0.113	0.060	0.077	NA	0.081	0.195	0.090	NA	NA	0.020	0.075	0.530
Italy	NA	0.165	0.033	0.048	NA	0.017	0.192	0.089	NA	NA	0.013	0.048	0.478
Latvia	NA	0.126	0.095	0.107	NA	0.029	0.206	0.109	NA	NA	0.023	0.078	0.563
Netherlands	NA	0.089	0.072	0.074	NA	0.204	0.183	0.090	NA	NA	0.036	0.082	0.590
Romania	NA	0.165	0.006	0.073	NA	0.031	0.197	0.073	NA	NA	0.016	0.083	0.499
Spain	NA	0.158	0.044	0.056	NA	0.065	0.186	0.074	NA	NA	0.021	0.036	0.495
Sweden	NA	0.122	0.091	0.056	NA	0.085	0.192	0.099	NA	NA	0.026	0.061	0.541
United Kingdom	NA	0.152	0.066	0.080	NA	0.100	0.195	0.075	NA	NA	0.017	0.067	0.552

SSB: sugar sweetened beverages, NA: not applicable.

ESM Table 19: Population attributable fractions for 12 food groups and stroke. Analyses based on theoretical minimum risk exposure level from disease-specific outcomes and including all associations (Scenario A).

Country	Refined grain	Whole grain	Vegetables	Fruit	Eggs	SSB	Nuts	Legumes	Processed meat	Red meat	Dairy	Fish	Total
Austria	0.032	0.035	0.054	0.087	0.022	0.019	0.062	0.088	0.043	0.038	0.029	0.090	0.461
Belgium	0.028	0.074	0.041	0.079	0.021	0.082	0.056	0.086	0.069	0.042	0.030	0.096	0.517
Czech Republic	0.022	0.071	0.034	0.081	0.018	0.042	0.062	0.099	0.130	0.064	0.026	0.090	0.537
Denmark	0.030	0.037	0.023	0.060	0.014	0.042	0.054	0.095	0.084	0.081	0.014	0.094	0.478
Finland	0.029	0.061	0.032	0.082	0.024	0.020	0.056	0.092	0.081	0.044	0.012	0.096	0.479
France	0.027	0.076	0.025	0.074	0.016	0.024	0.023	0.091	0.085	0.063	0.023	0.078	0.465
Germany	0.031	0.075	0.042	0.066	0.022	0.046	0.055	0.087	0.091	0.031	0.026	0.096	0.500
Hungary	0.020	0.079	0.022	0.058	0.016	0.024	0.055	0.085	0.108	0.057	0.020	0.107	0.492
Ireland	0.030	0.056	0.025	0.101	0.020	0.041	0.053	0.093	0.103	0.071	0.017	0.095	0.520
Italy	0.021	0.081	0.017	0.053	0.016	0.009	0.052	0.086	0.080	0.061	0.025	0.063	0.441
Latvia	0.027	0.062	0.072	0.131	0.025	0.015	0.070	0.084	0.099	0.073	0.029	0.098	0.559
Netherlands	0.032	0.046	0.038	0.096	0.023	0.102	0.055	0.085	0.079	0.059	0.016	0.103	0.534
Romania	0.025	0.081	0.030	0.087	0.014	0.016	0.053	0.089	0.091	0.067	0.028	0.104	0.509
Spain	0.029	0.077	0.022	0.063	0.012	0.033	0.053	0.078	0.081	0.079	0.012	0.048	0.455
Sweden	0.029	0.060	0.060	0.073	0.020	0.044	0.053	0.092	0.074	0.031	0.016	0.079	0.478
United Kingdom	0.027	0.074	0.031	0.103	0.020	0.051	0.053	0.084	0.078	0.035	0.021	0.086	0.498

SSB: sugar sweetened beverages.

ESM Table 20: Population attributable fractions for 12 food groups and stroke. Analyses based on theoretical minimum risk exposure level from disease-specific outcomes and including significant associations (Scenario B).

Country	Refined grain	Whole grain	Vegetables	Fruit	Eggs	SSB	Nuts	Legumes	Processed meat	Red meat	Dairy	Fish	Total
Austria	NA	NA	0.054	0.087	NA	0.019	NA	NA	0.043	0.038	0.029	0.090	0.310
Belgium	NA	NA	0.041	0.079	NA	0.082	NA	NA	0.069	0.042	0.030	0.096	0.366
Czech Republic	NA	NA	0.034	0.081	NA	0.042	NA	NA	0.130	0.064	0.026	0.090	0.386
Denmark	NA	NA	0.023	0.060	NA	0.042	NA	NA	0.084	0.081	0.014	0.094	0.339
Finland	NA	NA	0.032	0.082	NA	0.020	NA	NA	0.081	0.044	0.012	0.096	0.317
France	NA	NA	0.025	0.074	NA	0.024	NA	NA	0.085	0.063	0.023	0.078	0.318
Germany	NA	NA	0.042	0.066	NA	0.046	NA	NA	0.091	0.031	0.026	0.096	0.339
Hungary	NA	NA	0.022	0.058	NA	0.024	NA	NA	0.108	0.057	0.020	0.107	0.338
Ireland	NA	NA	0.025	0.101	NA	0.041	NA	NA	0.103	0.071	0.017	0.095	0.378
Italy	NA	NA	0.017	0.053	NA	0.009	NA	NA	0.080	0.061	0.025	0.063	0.271
Latvia	NA	NA	0.072	0.131	NA	0.015	NA	NA	0.099	0.073	0.029	0.098	0.419
Netherlands	NA	NA	0.038	0.096	NA	0.102	NA	NA	0.079	0.059	0.016	0.103	0.403
Romania	NA	NA	0.030	0.087	NA	0.016	NA	NA	0.091	0.067	0.028	0.104	0.356
Spain	NA	NA	0.022	0.063	NA	0.033	NA	NA	0.081	0.079	0.012	0.048	0.295
Sweden	NA	NA	0.060	0.073	NA	0.044	NA	NA	0.074	0.031	0.016	0.079	0.322
United Kingdom	NA	NA	0.031	0.103	NA	0.051	NA	NA	0.078	0.035	0.021	0.086	0.344

SSB: sugar sweetened beverages, NA: not applicable.

ESM Table 21: Population attributable fractions for 12 food groups and stroke. Analyses based on single theoretical minimum risk exposure level from combined outcomes and including all associations (Scenario C).

Country	Refined grain	Whole grain	Vegetables	Fruit	Eggs	SSB	Nuts	Legumes	Processed meat	Red meat	Dairy	Fish	Total
Austria	-0.003	0.035	0.031	0.077	-0.011	0.019	0.058	0.088	0.043	0.019	0.022	0.090	0.383
Belgium	-0.008	0.074	0.019	0.069	-0.012	0.082	0.052	0.086	0.069	0.023	0.023	0.095	0.447
Czech Republic	-0.014	0.071	0.011	0.070	-0.014	0.042	0.057	0.099	0.130	0.045	0.019	0.090	0.470
Denmark	-0.006	0.037	0.000	0.049	-0.019	0.042	0.049	0.095	0.084	0.063	0.007	0.093	0.403
Finland	-0.006	0.061	0.009	0.071	-0.009	0.020	0.052	0.092	0.081	0.025	0.005	0.095	0.404
France	-0.008	0.076	0.002	0.063	-0.017	0.024	0.019	0.091	0.085	0.044	0.016	0.078	0.388
Germany	-0.004	0.075	0.019	0.056	-0.010	0.046	0.051	0.087	0.091	0.012	0.019	0.095	0.428
Hungary	-0.015	0.079	-0.001	0.047	-0.016	0.024	0.051	0.085	0.108	0.038	0.013	0.107	0.419
Ireland	-0.006	0.056	0.002	0.091	-0.012	0.041	0.049	0.093	0.103	0.053	0.010	0.095	0.451
Italy	-0.014	0.081	-0.007	0.042	-0.016	0.009	0.048	0.086	0.080	0.043	0.018	0.062	0.361
Latvia	-0.008	0.062	0.050	0.121	-0.008	0.015	0.066	0.084	0.099	0.055	0.022	0.097	0.496
Netherlands	-0.004	0.046	0.015	0.085	-0.009	0.102	0.051	0.085	0.079	0.041	0.009	0.102	0.467
Romania	-0.010	0.081	0.006	0.077	-0.018	0.016	0.049	0.089	0.091	0.048	0.021	0.104	0.439
Spain	-0.006	0.077	-0.001	0.052	-0.021	0.033	0.049	0.078	0.081	0.061	0.005	0.048	0.376
Sweden	-0.006	0.060	0.037	0.063	-0.012	0.044	0.049	0.092	0.074	0.012	0.009	0.078	0.403
United Kingdom	-0.008	0.074	0.008	0.093	-0.012	0.051	0.049	0.084	0.078	0.016	0.015	0.086	0.426

SSB: sugar sweetened beverages.

ESM Table 22: Population attributable fractions for 12 food groups and stroke. Analyses based on single theoretical minimum risk exposure level from combined outcomes and including significant associations (Scenario D).

Country	Refined grain	Whole grain	Vegetables	Fruit	Eggs	SSB	Nuts	Legumes	Processed meat	Red meat	Dairy	Fish	Total
Austria	NA	NA	0.031	0.077	NA	0.019	NA	NA	0.043	0.038	0.022	0.090	0.281
Belgium	NA	NA	0.019	0.069	NA	0.082	NA	NA	0.069	0.042	0.023	0.096	0.338
Czech Republic	NA	NA	0.011	0.070	NA	0.042	NA	NA	0.130	0.064	0.019	0.090	0.359
Denmark	NA	NA	0.000	0.049	NA	0.042	NA	NA	0.084	0.081	0.007	0.094	0.310
Finland	NA	NA	0.009	0.071	NA	0.020	NA	NA	0.081	0.044	0.005	0.096	0.287
France	NA	NA	0.002	0.063	NA	0.024	NA	NA	0.085	0.063	0.016	0.078	0.289
Germany	NA	NA	0.019	0.056	NA	0.046	NA	NA	0.091	0.031	0.019	0.096	0.310
Hungary	NA	NA	-0.001	0.047	NA	0.024	NA	NA	0.108	0.057	0.013	0.107	0.310
Ireland	NA	NA	0.002	0.091	NA	0.041	NA	NA	0.103	0.071	0.010	0.095	0.351
Italy	NA	NA	-0.007	0.042	NA	0.009	NA	NA	0.080	0.061	0.018	0.063	0.240
Latvia	NA	NA	0.050	0.121	NA	0.015	NA	NA	0.099	0.073	0.022	0.098	0.394
Netherlands	NA	NA	0.015	0.085	NA	0.102	NA	NA	0.079	0.059	0.009	0.103	0.377
Romania	NA	NA	0.006	0.077	NA	0.016	NA	NA	0.091	0.067	0.021	0.104	0.328
Spain	NA	NA	-0.001	0.052	NA	0.033	NA	NA	0.081	0.079	0.005	0.048	0.265
Sweden	NA	NA	0.037	0.063	NA	0.044	NA	NA	0.074	0.031	0.009	0.079	0.292
United Kingdom	NA	NA	0.008	0.093	NA	0.051	NA	NA	0.078	0.035	0.015	0.086	0.316

SSB: sugar sweetened beverages, NA: not applicable.

ESM Table 23: Population attributable fractions for 12 food groups and type 2 diabetes. Analyses based on theoretical minimum risk exposure level from disease-specific outcomes and including all associations (Scenario A).

Country	Refined grain	Whole grain	Vegetables	Fruit	Eggs	SSB	Nuts	Legumes	Processed meat	Red meat	Dairy	Fish	Total
Austria	0.023	0.087	0.053	0.050	0.006	0.041	0.013	0.053	0.086	0.068	0.040	0.012	0.422
Belgium	0.038	0.221	0.044	0.046	0.028	0.123	0.012	0.051	0.135	0.074	0.042	0.010	0.585
Czech Republic	0.057	0.200	0.039	0.047	0.046	0.084	0.013	0.056	0.236	0.108	0.037	0.012	0.635
Denmark	0.033	0.087	0.028	0.038	0.022	0.076	0.012	0.055	0.165	0.137	0.020	0.011	0.513
Finland	0.033	0.177	0.037	0.048	0.032	0.042	0.012	0.055	0.157	0.075	0.017	0.010	0.520
France	0.039	0.229	0.032	0.043	0.019	0.055	0.005	0.049	0.165	0.109	0.033	0.016	0.574
Germany	0.028	0.226	0.044	0.041	0.014	0.078	0.012	0.052	0.172	0.055	0.037	0.010	0.562
Hungary	0.060	0.234	0.030	0.037	0.054	0.045	0.012	0.045	0.202	0.099	0.029	0.007	0.602
Ireland	0.032	0.156	0.032	0.056	0.016	0.087	0.011	0.053	0.195	0.121	0.025	0.011	0.572
Italy	0.058	0.244	0.018	0.034	0.043	0.026	0.011	0.050	0.157	0.106	0.035	0.020	0.579
Latvia	0.037	0.176	0.064	0.068	0.023	0.033	0.014	0.051	0.188	0.122	0.041	0.010	0.585
Netherlands	0.025	0.128	0.043	0.053	0.011	0.155	0.012	0.049	0.154	0.102	0.022	0.008	0.554
Romania	0.046	0.244	0.022	0.050	0.077	0.037	0.011	0.048	0.176	0.114	0.039	0.008	0.609
Spain	0.032	0.231	0.025	0.039	0.053	0.072	0.011	0.043	0.157	0.132	0.018	0.023	0.593
Sweden	0.032	0.163	0.059	0.044	0.019	0.092	0.011	0.054	0.145	0.056	0.022	0.016	0.528
United Kingdom	0.040	0.222	0.038	0.056	0.019	0.102	0.012	0.046	0.154	0.064	0.031	0.014	0.573

SSB: sugar sweetened beverages.

ESM Table 24: Population attributable fractions for 12 food groups and type 2 diabetes. Analyses based on theoretical minimum risk exposure level from disease-specific outcomes and including significant associations (Scenario B).

**Country**	**Refined grain**	**Whole grain**	**Vegetables**	**Fruit**	**Eggs**	**SSB**	**Nuts**	**Legumes**	**Processed meat**	**Red meat**	**Dairy**	**Fish**	**Total**
Austria	0.023	0.087	0.053	0.050	0.006	0.041	NA	NA	0.086	0.068	0.040	NA	0.374
Belgium	0.038	0.221	0.044	0.046	0.028	0.123	NA	NA	0.135	0.074	0.042	NA	0.553
Czech Republic	0.057	0.200	0.039	0.047	0.046	0.084	NA	NA	0.236	0.108	0.037	NA	0.603
Denmark	0.033	0.087	0.028	0.038	0.022	0.076	NA	NA	0.165	0.137	0.020	NA	0.473
Finland	0.033	0.177	0.037	0.048	0.032	0.042	NA	NA	0.157	0.075	0.017	NA	0.480
France	0.039	0.229	0.032	0.043	0.019	0.055	NA	NA	0.165	0.109	0.033	NA	0.542
Germany	0.028	0.226	0.044	0.041	0.014	0.078	NA	NA	0.172	0.055	0.037	NA	0.528
Hungary	0.060	0.234	0.030	0.037	0.054	0.045	NA	NA	0.202	0.099	0.029	NA	0.576
Ireland	0.032	0.156	0.032	0.056	0.016	0.087	NA	NA	0.195	0.121	0.025	NA	0.538
Italy	0.058	0.244	0.018	0.034	0.043	0.026	NA	NA	0.157	0.106	0.035	NA	0.542
Latvia	0.037	0.176	0.064	0.068	0.023	0.033	NA	NA	0.188	0.122	0.041	NA	0.552
Netherlands	0.025	0.128	0.043	0.053	0.011	0.155	NA	NA	0.154	0.102	0.022	NA	0.522
Romania	0.046	0.244	0.022	0.050	0.077	0.037	NA	NA	0.176	0.114	0.039	NA	0.582
Spain	0.032	0.231	0.025	0.039	0.053	0.072	NA	NA	0.157	0.132	0.018	NA	0.559
Sweden	0.032	0.163	0.059	0.044	0.019	0.092	NA	NA	0.145	0.056	0.022	NA	0.487
United Kingdom	0.040	0.222	0.038	0.056	0.019	0.102	NA	NA	0.154	0.064	0.031	NA	0.541

SSB: sugar sweetened beverages, NA: not applicable.

ESM Table 25: Population attributable fractions for 12 food groups and type 2 diabetes. Analyses based on single theoretical minimum risk exposure level from combined outcomes and including all associations (Scenario C).

Country	Refined grain	Whole grain	Vegetables	Fruit	Eggs	SSB	Nuts	Legumes	Processed meat	Red meat	Dairy	Fish	Total
Austria	0.011	0.053	0.046	0.043	0.006	0.041	0.013	0.053	0.086	0.032	0.030	-0.021	0.332
Belgium	0.026	0.193	0.037	0.040	0.028	0.123	0.011	0.051	0.135	0.037	0.031	-0.023	0.521
Czech Republic	0.045	0.171	0.032	0.040	0.046	0.084	0.012	0.056	0.236	0.073	0.026	-0.021	0.578
Denmark	0.021	0.054	0.021	0.031	0.022	0.076	0.011	0.055	0.165	0.103	0.009	-0.022	0.437
Finland	0.021	0.147	0.030	0.041	0.032	0.042	0.011	0.055	0.157	0.039	0.006	-0.023	0.445
France	0.027	0.200	0.025	0.037	0.019	0.055	0.004	0.049	0.165	0.074	0.022	-0.017	0.507
Germany	0.016	0.198	0.038	0.035	0.014	0.078	0.011	0.052	0.172	0.018	0.026	-0.023	0.494
Hungary	0.049	0.206	0.023	0.030	0.054	0.045	0.011	0.045	0.202	0.063	0.018	-0.026	0.540
Ireland	0.020	0.125	0.026	0.050	0.016	0.087	0.011	0.053	0.195	0.087	0.014	-0.022	0.505
Italy	0.046	0.216	0.011	0.028	0.043	0.026	0.011	0.050	0.157	0.071	0.024	-0.013	0.513
Latvia	0.025	0.145	0.058	0.062	0.023	0.033	0.013	0.051	0.188	0.087	0.030	-0.023	0.521
Netherlands	0.013	0.096	0.036	0.047	0.011	0.155	0.011	0.049	0.154	0.066	0.011	-0.025	0.485
Romania	0.034	0.216	0.015	0.043	0.077	0.037	0.011	0.048	0.176	0.080	0.029	-0.025	0.548
Spain	0.020	0.203	0.018	0.033	0.053	0.072	0.011	0.043	0.157	0.098	0.007	-0.009	0.529
Sweden	0.020	0.132	0.052	0.037	0.019	0.092	0.011	0.054	0.145	0.019	0.011	-0.017	0.454
United Kingdom	0.028	0.193	0.031	0.050	0.019	0.102	0.011	0.046	0.154	0.027	0.020	-0.019	0.507

SSB: sugar sweetened beverages.

ESM Table 26: Population attributable fractions for 12 food groups and type 2 diabetes. Analyses based on single theoretical minimum risk exposure level from combined outcomes and including significant associations (Scenario D).

Country	Refined grain	Whole grain	Vegetables	Fruit	Eggs	SSB	Nuts	Legumes	Processed meat	Red meat	Dairy	Fish	Total
Austria	0.023	0.054	0.046	0.043	0.006	0.041	NA	NA	0.086	0.068	0.030	NA	0.335
Belgium	0.038	0.193	0.037	0.040	0.028	0.123	NA	NA	0.135	0.074	0.031	NA	0.525
Czech Republic	0.057	0.171	0.032	0.040	0.046	0.084	NA	NA	0.236	0.108	0.026	NA	0.579
Denmark	0.033	0.054	0.021	0.031	0.022	0.076	NA	NA	0.165	0.137	0.009	NA	0.440
Finland	0.033	0.147	0.030	0.041	0.032	0.042	NA	NA	0.157	0.075	0.006	NA	0.448
France	0.039	0.201	0.025	0.037	0.019	0.055	NA	NA	0.165	0.109	0.022	NA	0.514
Germany	0.028	0.198	0.038	0.035	0.014	0.078	NA	NA	0.172	0.055	0.026	NA	0.499
Hungary	0.060	0.206	0.023	0.030	0.054	0.045	NA	NA	0.202	0.099	0.018	NA	0.549
Ireland	0.032	0.126	0.026	0.050	0.016	0.087	NA	NA	0.195	0.121	0.014	NA	0.509
Italy	0.058	0.216	0.011	0.028	0.043	0.026	NA	NA	0.157	0.106	0.024	NA	0.514
Latvia	0.037	0.146	0.058	0.062	0.023	0.033	NA	NA	0.188	0.122	0.030	NA	0.525
Netherlands	0.025	0.096	0.036	0.047	0.011	0.155	NA	NA	0.154	0.102	0.011	NA	0.492
Romania	0.046	0.216	0.015	0.043	0.077	0.037	NA	NA	0.176	0.114	0.029	NA	0.556
Spain	0.032	0.203	0.018	0.033	0.053	0.072	NA	NA	0.157	0.132	0.007	NA	0.532
Sweden	0.032	0.133	0.052	0.037	0.019	0.092	NA	NA	0.145	0.056	0.011	NA	0.455
United Kingdom	0.040	0.194	0.031	0.050	0.019	0.102	NA	NA	0.154	0.064	0.020	NA	0.513

SSB: sugar sweetened beverages, NA: not applicable.

ESM Table 27: Population attributable fractions for 12 food groups and colorectal cancer. Analyses based on theoretical minimum risk exposure level from disease-specific outcomes and including all associations (Scenario A).

Country	Refined grain	Whole grain	Vegetables	Fruit	Eggs	SSB	Nuts	Legumes	Processed meat	Red meat	Dairy	Fish	Total
Austria	NA	0.167	0.064	0.045	-0.110	NA	0.064	0.016	0.074	0.036	0.182	0.036	0.464
Belgium	NA	0.242	0.056	0.044	-0.087	NA	0.070	0.015	0.113	0.043	0.183	0.040	0.546
Czech Republic	NA	0.255	0.051	0.044	-0.063	NA	0.074	0.017	0.290	0.068	0.172	0.037	0.655
Denmark	NA	0.180	0.040	0.034	-0.059	NA	0.072	0.016	0.101	0.085	0.114	0.038	0.484
Finland	NA	0.217	0.048	0.043	-0.099	NA	0.069	0.016	0.138	0.050	0.095	0.040	0.488
France	NA	0.259	0.045	0.041	-0.071	NA	0.036	0.014	0.114	0.064	0.162	0.030	0.534
Germany	NA	0.245	0.056	0.036	-0.104	NA	0.061	0.015	0.154	0.033	0.171	0.040	0.543
Hungary	NA	0.267	0.043	0.033	-0.047	NA	0.067	0.013	0.194	0.058	0.147	0.045	0.596
Ireland	NA	0.211	0.045	0.053	-0.094	NA	0.073	0.016	0.149	0.075	0.135	0.039	0.534
Italy	NA	0.269	0.026	0.031	-0.055	NA	0.071	0.015	0.098	0.063	0.169	0.023	0.543
Latvia	NA	0.230	0.073	0.063	-0.110	NA	0.075	0.014	0.153	0.081	0.183	0.041	0.586
Netherlands	NA	0.166	0.055	0.050	-0.111	NA	0.066	0.014	0.126	0.064	0.121	0.043	0.473
Romania	NA	0.270	0.007	0.046	-0.021	NA	0.073	0.014	0.134	0.070	0.181	0.044	0.593
Spain	NA	0.263	0.033	0.035	-0.025	NA	0.068	0.012	0.128	0.086	0.112	0.017	0.549
Sweden	NA	0.230	0.070	0.042	-0.092	NA	0.071	0.016	0.107	0.030	0.124	0.030	0.497
United Kingdom	NA	0.255	0.050	0.054	-0.092	NA	0.072	0.014	0.098	0.031	0.154	0.034	0.523

SSB: sugar sweetened beverages, NA: not applicable.

ESM Table 28: Population attributable fractions for 12 food groups and colorectal cancer. Analyses based on theoretical minimum risk exposure level from disease-specific outcomes and including significant associations (Scenario B).

**Country**	**Refined grain**	**Whole grain**	**Vegetables**	**Fruit**	**Eggs**	**SSB**	**Nuts**	**Legumes**	**Processed meat**	**Red meat**	**Dairy**	**Fish**	**Total**
Austria	NA	0.167	0.064	0.045	NA	NA	NA	NA	0.074	0.036	0.182	NA	0.456
Belgium	NA	0.242	0.056	0.044	NA	NA	NA	NA	0.113	0.043	0.183	NA	0.526
Czech Republic	NA	0.255	0.051	0.044	NA	NA	NA	NA	0.290	0.068	0.172	NA	0.630
Denmark	NA	0.180	0.040	0.034	NA	NA	NA	NA	0.101	0.085	0.114	NA	0.446
Finland	NA	0.217	0.048	0.043	NA	NA	NA	NA	0.138	0.050	0.095	NA	0.471
France	NA	0.259	0.045	0.041	NA	NA	NA	NA	0.114	0.064	0.162	NA	0.528
Germany	NA	0.245	0.056	0.036	NA	NA	NA	NA	0.154	0.033	0.171	NA	0.534
Hungary	NA	0.267	0.043	0.033	NA	NA	NA	NA	0.194	0.058	0.147	NA	0.561
Ireland	NA	0.211	0.045	0.053	NA	NA	NA	NA	0.149	0.075	0.135	NA	0.514
Italy	NA	0.269	0.026	0.031	NA	NA	NA	NA	0.098	0.063	0.169	NA	0.515
Latvia	NA	0.230	0.073	0.063	NA	NA	NA	NA	0.153	0.081	0.183	NA	0.574
Netherlands	NA	0.166	0.055	0.050	NA	NA	NA	NA	0.126	0.064	0.121	NA	0.462
Romania	NA	0.270	0.007	0.046	NA	NA	NA	NA	0.134	0.070	0.181	NA	0.544
Spain	NA	0.263	0.033	0.035	NA	NA	NA	NA	0.128	0.086	0.112	NA	0.514
Sweden	NA	0.230	0.070	0.042	NA	NA	NA	NA	0.107	0.030	0.124	NA	0.480
United Kingdom	NA	0.255	0.050	0.054	NA	NA	NA	NA	0.098	0.031	0.154	NA	0.505

SSB: sugar sweetened beverages, NA: not applicable.

ESM Table 29: Population attributable fractions for 12 food groups and colorectal cancer. Analyses based on single theoretical minimum risk exposure level from combined outcomes and including all associations (Scenario C).

**Country**	**Refined grain**	**Whole grain**	**Vegetables**	**Fruit**	**Eggs**	**SSB**	**Nuts**	**Legumes**	**Processed meat**	**Red meat**	**Dairy**	**Fish**	**Total**
Austria	NA	0.078	0.062	0.036	0.045	NA	0.053	0.016	0.074	0.024	0.090	0.036	0.412
Belgium	NA	0.161	0.054	0.034	0.065	NA	0.059	0.015	0.113	0.031	0.092	0.040	0.502
Czech Republic	NA	0.176	0.049	0.034	0.085	NA	0.064	0.017	0.290	0.056	0.080	0.037	0.621
Denmark	NA	0.093	0.038	0.024	0.089	NA	0.061	0.016	0.101	0.074	0.015	0.038	0.434
Finland	NA	0.133	0.046	0.033	0.054	NA	0.058	0.016	0.138	0.038	-0.006	0.040	0.439
France	NA	0.180	0.043	0.031	0.079	NA	0.025	0.014	0.114	0.052	0.068	0.030	0.488
Germany	NA	0.164	0.054	0.027	0.050	NA	0.050	0.015	0.154	0.021	0.078	0.039	0.498
Hungary	NA	0.188	0.041	0.023	0.099	NA	0.056	0.013	0.194	0.047	0.052	0.045	0.556
Ireland	NA	0.126	0.043	0.043	0.059	NA	0.062	0.016	0.149	0.064	0.038	0.039	0.488
Italy	NA	0.191	0.024	0.021	0.093	NA	0.061	0.015	0.098	0.051	0.076	0.023	0.498
Latvia	NA	0.148	0.071	0.053	0.045	NA	0.064	0.014	0.153	0.069	0.092	0.041	0.546
Netherlands	NA	0.078	0.053	0.041	0.044	NA	0.055	0.014	0.126	0.052	0.023	0.043	0.422
Romania	NA	0.193	0.005	0.036	0.122	NA	0.063	0.014	0.134	0.058	0.089	0.044	0.554
Spain	NA	0.185	0.031	0.026	0.118	NA	0.057	0.012	0.128	0.074	0.013	0.017	0.505
Sweden	NA	0.148	0.068	0.032	0.061	NA	0.060	0.016	0.107	0.018	0.027	0.030	0.448
United Kingdom	NA	0.176	0.048	0.044	0.061	NA	0.062	0.014	0.098	0.019	0.060	0.034	0.476

SSB: sugar sweetened beverages, NA: not applicable.

ESM Table 30: Population attributable fractions for 12 food groups and colorectal cancer. Analyses based on single theoretical minimum risk exposure level from combined outcomes and including significant associations (Scenario D).

Country	Refined grain	Whole grain	Vegetables	Fruit	Eggs	SSB	Nuts	Legumes	Processed meat	Red meat	Dairy	Fish	Total
Austria	NA	0.077	0.062	0.036	NA	NA	NA	NA	0.074	0.036	0.090	NA	0.322
Belgium	NA	0.160	0.054	0.034	NA	NA	NA	NA	0.113	0.043	0.092	NA	0.409
Czech Republic	NA	0.175	0.049	0.034	NA	NA	NA	NA	0.290	0.068	0.080	NA	0.539
Denmark	NA	0.092	0.038	0.024	NA	NA	NA	NA	0.101	0.085	0.015	NA	0.309
Finland	NA	0.133	0.046	0.033	NA	NA	NA	NA	0.138	0.050	-0.006	NA	0.341
France	NA	0.179	0.043	0.031	NA	NA	NA	NA	0.114	0.064	0.068	NA	0.411
Germany	NA	0.164	0.054	0.027	NA	NA	NA	NA	0.154	0.033	0.078	NA	0.419
Hungary	NA	0.188	0.041	0.023	NA	NA	NA	NA	0.194	0.058	0.052	NA	0.453
Ireland	NA	0.126	0.043	0.043	NA	NA	NA	NA	0.149	0.075	0.038	NA	0.395
Italy	NA	0.190	0.024	0.021	NA	NA	NA	NA	0.098	0.063	0.076	NA	0.396
Latvia	NA	0.148	0.071	0.053	NA	NA	NA	NA	0.153	0.081	0.092	NA	0.469
Netherlands	NA	0.077	0.053	0.041	NA	NA	NA	NA	0.126	0.064	0.023	NA	0.330
Romania	NA	0.192	0.005	0.036	NA	NA	NA	NA	0.134	0.070	0.089	NA	0.431
Spain	NA	0.184	0.031	0.026	NA	NA	NA	NA	0.128	0.086	0.013	NA	0.394
Sweden	NA	0.147	0.068	0.032	NA	NA	NA	NA	0.107	0.030	0.027	NA	0.352
United Kingdom	NA	0.175	0.048	0.044	NA	NA	NA	NA	0.098	0.031	0.060	NA	0.383

SSB: sugar sweetened beverages, NA: not applicable.

ESM Table 31: Disability-adjusted life years for individual outcomes attributable to 12 food groups in 16 European countries using disease-specific theoretical minimum risk exposure levels for all outcomes including all associations (Scenario A).

**Country**	**Coronary heart disease**	**Stroke**	**Type 2 diabetes**	**Colorectal cancer**	**Total**
Austria	133 830<br>(123 915-144 164)	31 709<br>(27 877-35 795)	23 696<br>(19 727-28 009)	17 995<br>(16 323-19 735)	207 229<br>(195 640-219 199)
Belgium	155 014<br>(141 868-168 711)	60 171<br>(53 448-67 253)	36 230<br>(28 325-45 133)	31 288<br>(27 946-34 813)	282 703<br>(265 375-300 634)
Czech Republic	339 488<br>(318 686-360 676)	88 701<br>(80 353-97 455)	64 566<br>(51 348-79 202)	50 993<br>(45 619-56 611)	543 748<br>(516 978-571 320)
Denmark	61 446<br>(54 298-69 045)	28 683<br>(25 467-32 100)	20 295<br>(16 661-24 288)	17 088<br>(14 961-19 361)	127 512<br>(118 508-136 847)
Finland	100 288<br>(90 509-110 489)	35 955<br>(31 721-40 439)	17 599<br>(13 058-22 839)	10 023<br>(8 916-11 203)	163 865<br>(152 040-176 136)
France	553 334<br>(513 558-594 422)	255 189<br>(230 349-281 247)	195 162<br>(159 704-233 858)	175 877<br>(160 654-191 837)	1 179 561<br>(1 117 898-1 243 006)
Germany	1 630 560<br>(1 495 313-1 771 985)	462 871<br>(411 889-516 921)	377 393<br>(304 328-458 309)	263 940<br>(238 188-290 759)	2 734 764<br>(2 569 376-2 905 901)
Hungary	389 159<br>(352 095-428 730)	114 500<br>(102 648-127 106)	53 320<br>(43 301-64 230)	59 726<br>(53 138-66 614)	616 706<br>(575 581-659 541)
Ireland	56 962<br>(50 435-63 882)	16 463<br>(14 382-18 693)	12 107<br>(9 258-15 348)	10 748<br>(9 271-12 338)	96 279<br>(88 608-104 287)
Italy	878 773<br>(807 177-953 541)	282 969<br>(253 452-314 086)	295 875<br>(243 138-353 908)	181 420<br>(162 668-201 104)	1 639 038<br>(1 542 420-1 739 077)
Latvia	101 281	40 889	9 214	6 940	158 323

	(90 366-112 887)	(36 137-45 975)	(7 347-11 290)	(5 991-7 959)	(146 106-171 139)
Netherlands	177 601<br>(161 761-194 089)	86 600<br>(78 071-95 525)	66 461<br>(51 449-83 503)	49 705<br>(44 817-54 869)	380 367<br>(356 120-405 845)
Romania	711 996<br>(647 448-779 078)	395 709<br>(358 876-434 770)	62 194<br>(49 657-76 084)	69 335<br>(61 963-77 149)	1 239 234<br>(1 162 976-1 318 482)
Spain	480 747<br>(450 503-512 054)	177 065<br>(160 009-194 944)	154 191<br>(121 904-190 272)	137 690<br>(125 683-150 236)	949 693<br>(900 083-1 001 433)
Sweden	153 434<br>(136 450-171 454)	49 349<br>(43 487-55 571)	40 619<br>(31 220-51 279)	25 695<br>(22 690-28 878)	269 096<br>(248 244-290 950)
United Kingdom	846 567<br>(794 621-899 658)	332 723<br>(307 878-358 269)	161 036<br>(122 235-204 708)	165 299<br>(155 206-175 695)	1 505 625<br>(1 434 794-1 578 942)
**TOTAL**	**6 770 479**<br>**(6 581 069-6 966 069)**	**2 459 545**<br>**(2 375 790-2 545 402)**	**1 589 958**<br>**(1 473 245-1 712 124)**	**1 273 761**<br>**(1 232 241-1 315 757)**	**12 093 744**<br>**(11 850 947-12 340 261)**
**Proportion**	**67% (64%-70%)**	**49% (46%-53%)**	**57% (50%-66%)**	**54% (51%-57%)**	**59% (55%-65%)**

ESM Table 32. Disability-adjusted life years for individual outcomes attributable to 12 food groups in 16 European countries using disease-specific theoretical minimum risk exposure levels for all outcomes including significant associations (Scenario B).

**Country**	**Coronary heart disease**	**Stroke**	**Type 2 diabetes**	**Colorectal cancer**	**Total**
Austria	116 086 (107 485-125 050)	21 365 (18 783-24 118)	20 978 (17 464-24 796)	17 685 (16 042-19 395)	176 113 (166 232-186 343)
Belgium	132 187 (120 977-143 867)	42 549 (37 795-47 557)	34 226 (26 758-42 636)	30 102 (26 886-33 493)	239 063 (224 210-254 454)
Czech Republic	246 866 (231 739-262 273)	63 733 (57 735-70 023)	61 361 (48 799-75 270)	49 040 (43 872-54 444)	421 000 (399 569-443 184)
Denmark	49 422 (43 672-55 533)	20 329 (18 049-22 751)	18 699 (15 351-22 378)	15 721 (13 765-17 812)	104 171 (96 795-111 836)
Finland	80 676 (72 809-88 882)	23 773 (20 973-26 738)	16 267 (12 070-21 111)	9 662 (8 594-10 799)	130 378 (120 803-140 319)
France	413 691 (383 954-444 410)	174 775 (157 763-192 622)	184 459 (150 945-221 033)	173 917 (158 864-189 699)	946 842 (895 940-999 595)
Germany	1 378 436 (1 264 102-1 497 994)	313 558 (279 022-350 173)	354 326 (285 727-430 295)	259 572 (234 246-285 946)	2 305 892 (2 164 267-2 452 384)
Hungary	283 309 (256 326-312 117)	78 719 (70 570-87 385)	50 945 (41 372-61 369)	56 213 (50 013-62 696)	469 186 (438 427-501 146)
Ireland	45 883 (40 626-51 458)	11 953 (10 443-13 573)	11 385 (8 705-14 432)	10 354 (8 930-11 885)	79 574 (73 229-86 209)
Italy	633 350 (581 749-687 236)	173 949 (155 804-193 077)	277 364 (227 927-331 766)	172 146 (154 352-190 823)	1 256 809 (1 180 165-1 336 768)
Latvia	78 976	30 602	8 694	6 795	125 066

	(70 465-88 026)	(27 045-34 408)	(6 932-10 653)	(5 866-7 793)	(115 516-135 068)
Netherlands	152 810<br>(139 181-166 997)	65 296<br>(58 865-72 025)	62 545<br>(48 417-78 582)	48 535<br>(43 763-53 578)	329 186<br>(307 663-351 843)
Romania	525 030<br>(477 431-574 497)	276 633<br>(250 884-303 940)	59 376<br>(47 407-72 636)	63 544<br>(56 789-70 706)	924 583<br>(868 377-983 107)
Spain	367 743<br>(344 608-391 691)	114 804<br>(103 745-126 396)	145 535<br>(115 060-179 590)	128 821<br>(117 587-140 559)	756 903<br>(715 098-801 104)
Sweden	130 300<br>(115 877-145 603)	33 176<br>(29 235-37 359)	37 465<br>(28 796-47 298)	24 819<br>(21 917-27 894)	225 760<br>(207 925-244 468)
United Kingdom	710 485<br>(666 889-755 042)	229 707<br>(212 555-247 344)	152 023<br>(115 393-193 251)	159 836<br>(150 077-169 889)	1 252 051<br>(1 191 084-1 315 259)
**TOTAL**	**5 345 249**<br>**(5 192 897-5 502 552)**	**1 674 920**<br>**(1 618 288-1 733 074)**	**1 495 646**<br>**(1 385 841-1 610 537)**	**1 226 763**<br>**(1 186 624-1 267 389)**	**9 742 578**<br>**(9 540 578-9 947 540)**
**Proportion**	**53% (50%-55%)**	**33% (31%-36%)**	**54% (47%-62%)**	**52% (49%-55%)**	**48% (44%-52%)**

ESM Table 33. Disability-adjusted life years for individual outcomes attributable to 12 food groups in 16 European countries using single theoretical minimum risk exposure levels for all outcomes including all associations (Scenario C).

**Country**	**Coronary heart disease**	**Stroke**	**Type 2 diabetes**	**Colorectal cancer**	**Total**
Austria	131 444<br>(121 706-141 595)	26 365<br>(23 179-29 762)	18 633<br>(15 511-22 024)	15 973<br>(14 489-17 517)	192 414<br>(181 496-203 670)
Belgium	153 463<br>(140 448-167 023)	52 081<br>(46 262-58 210)	32 225<br>(25 194-40 143)	28 758<br>(25 686-31 998)	266 526<br>(250 170-283 445)
Czech Republic	336 921<br>(316 277-357 949)	77 689<br>(70 378-85 356)	58 767<br>(46 736-72 089)	48 378<br>(43 279-53 708)	521 756<br>(496 114-548 206)
Denmark	60 602<br>(53 553-68 097)	24 172<br>(21 461-27 051)	17 290<br>(14 194-20 692)	15 317<br>(13 411-17 355)	117 382<br>(108 926-126 181)
Finland	99 090<br>(89 428-109 169)	30 330<br>(26 758-34 113)	15 062<br>(11 176-19 547)	9 001<br>(8 006-10 060)	153 483<br>(142 281-165 093)
France	544 692<br>(505 537-585 139)	212 931<br>(192 205-234 674)	172 545<br>(141 196-206 757)	160 914<br>(146 987-175 517)	1 091 083<br>(1 034 119-1 149 343)
Germany	1 610 107<br>(1 476 557-1 749 758)	396 208<br>(352 568-442 474)	331 552<br>(267 362-402 638)	242 292<br>(218 652-266 911)	2 580 159<br>(2 422 671-2 743 281)
Hungary	385 407<br>(348 700-424 596)	97 480<br>(87 389-108 212)	47 829<br>(38 841-57 615)	55 781<br>(49 628-62 214)	586 496<br>(546 508-628 188)
Ireland	56 264<br>(49 817-63 100)	14 275<br>(12 472-16 210)	10 694<br>(8 177-13 557)	9 834<br>(8 482-11 289)	91 067<br>(83 696-98 752)
Italy	868 284<br>(797 542-942 158)	231 369<br>(207 235-256 812)	262 238<br>(215 497-313 674)	166 544<br>(149 329-184 614)	1 528 435<br>(1 437 801-1 622 275)
Latvia	100 365	36 254	8 195	6 463	151 277

	(89 549-111 866)	(32 040-40 763)	(6 535-10 043)	(5 579-7 412)	(139 405-163 768)
Netherlands	175 672<br>(160 004-191 982)	75 726<br>(68 268-83 530)	58 121<br>(44 992-73 025)	44 321<br>(39 963-48 926)	353 840<br>(331 401-377 391)
Romania	704 013<br>(640 189-770 344)	340 855<br>(309 128-374 501)	55 971<br>(44 688-68 470)	64 707<br>(57 827-71 999)	1 165 546<br>(1 092 791-1 241 549)
Spain	474 584<br>(444 727-505 489)	146 517<br>(132 404-161 312)	137 661<br>(108 835-169 874)	126 675<br>(115 628-138 217)	885 437<br>(839 554-933 107)
Sweden	151 367<br>(134 612-169 145)	41 605<br>(36 663-46 852)	34 955<br>(26 866-44 128)	23 161<br>(20 453-26 031)	251 088<br>(231 309-271 729)
United Kingdom	836 132<br>(784 826-888 568)	284 544<br>(263 297-306 391)	142 327<br>(108 034-180 926)	150 595<br>(141 400-160 066)	1 413 598<br>(1 347 220-1 482 312)
**TOTAL**	**6 688 407**<br>**(6 501 304-6 881 635)**	**2 088 402**<br>**(2 017 264-2 161 436)**	**1 404 065**<br>**(1 301 004-1 511 977)**	**1 168 715**<br>**(1 130 580-1 207 275)**	**11 349 588**<br>**(11 119 976-11 583 653)**
**Proportion**	**66% (63%-69%)**	**41% (39%-45%)**	**50% (44%-58%)**	**50% (47%-53%)**	**56% (51%-61%)**

ESM Table 34. Disability-adjusted life years for individual outcomes attributable to 12 food groups in 16 European countries using single theoretical minimum risk exposure levels for all outcomes including significant associations (Scenario D).

**Country**	**Coronary heart disease**	**Stroke**	**Type 2 diabetes**	**Colorectal cancer**	**Total**
Austria	114 911<br>(106 398-123 785)	19 323<br>(16 988-21 813)	18 792<br>(15 645-22 213)	12 495<br>(11 334-13 703)	165 522<br>(155 999-175 383)
Belgium	131 309<br>(120 173-142 911)	39 374<br>(34 974-44 008)	32 506<br>(25 414-40 494)	23 411<br>(20 910-26 049)	226 600<br>(212 250-241 464)
Czech Republic	244 858<br>(229 855-260 140)	59 370<br>(53 782-65 229)	58 853<br>(46 804-72 194)	41 946<br>(37 526-46 569)	405 027<br>(384 288-426 519)
Denmark	48 948<br>(43 254-55 001)	18 622<br>(16 534-20 841)	17 403<br>(14 287-20 827)	10 908<br>(9 550-12 358)	95 881<br>(88 862-103 168)
Finland	79 978<br>(72 179-88 113)	21 568<br>(19 028-24 258)	15 174<br>(11 259-19 693)	6 988<br>(6 216-7 811)	123 709<br>(114 448-133 328)
France	408 674<br>(379 297-439 021)	158 691<br>(143 245-174 896)	174 788<br>(143 031-209 444)	135 594<br>(123 858-147 899)	877 747<br>(829 741-927 513)
Germany	1 367 356<br>(1 253 940-1 485 952)	287 219<br>(255 584-320 759)	334 639<br>(269 851-406 387)	203 747<br>(183 868-224 450)	2 192 961<br>(2 055 578-2 335 109)
Hungary	280 674<br>(253 942-309 213)	72 095<br>(64 632-80 032)	48 611<br>(39 477-58 557)	45 370<br>(40 365-50 602)	446 749<br>(416 785-477 968)
Ireland	45 480<br>(40 269-51 006)	11 106<br>(9 703-12 611)	10 777<br>(8 241-13 662)	7 945<br>(6 853-9 120)	75 308<br>(69 175-81 737)
Italy	626 493<br>(575 451-679 796)	153 844<br>(137 796-170 761)	262 820<br>(215 975-314 369)	132 239<br>(118 570-146 586)	1 175 395<br>(1 102 285-1 251 624)
Latvia	78 368	28 774	8 256	5 554	120 953

	(69 922-87 349)	(25 430-32 354)	(6 583-10 117)	(4 795-6 370)	(111 591-130 792)
Netherlands	151 755<br>(138 220-165 844)	61 131<br>(55 110-67 431)	58 980<br>(45 657-74 104)	34 632<br>(31 227-38 230)	306 498<br>(285 990-328 111)
Romania	519 809<br>(472 685-568 785)	255 123<br>(231 376-280 307)	56 722<br>(45 288-69 390)	50 414<br>(45 054-56 096)	882 069<br>(827 498-938 979)
Spain	364 030<br>(341 128-387 736)	102 999<br>(93 078-113 400)	138 415<br>(109 431-170 804)	98 779<br>(90 165-107 779)	704 223<br>(664 608-746 152)
Sweden	129 203<br>(114 902-144 378)	30 167<br>(26 584-33 971)	35 014<br>(26 911-44 203)	18 196<br>(16 069-20 450)	212 581<br>(195 346-230 635)
United Kingdom	704 756<br>(661 512-748 954)	210 882<br>(195 136-227 074)	144 013<br>(109 314-183 069)	121 304<br>(113 898-128 934)	1 180 957<br>(1 122 121-1 241 751)
**TOTAL**	**5 296 603<br>(5 145 644-5 452 525)**	**1 530 289<br>(1 478 557-1 583 413)**	**1 415 765<br>(1 311 873-1 524 514)**	**949 523<br>(918 299-981 176)**	**9 192 180<br>(8 997 492-9 390 309)**
**Proportions**	**52% (50%-55%)**	**30% (29%-33%)**	**51% (45%-59%)**	**40% (38%-43%)**	**45% (42%-49%)**
